# Ornaments Reveal Resistance of North European Cultures to the Spread of Farming

**DOI:** 10.1371/journal.pone.0121166

**Published:** 2015-04-08

**Authors:** Solange Rigaud, Francesco d'Errico, Marian Vanhaeren

**Affiliations:** 1 Centre National de la Recherche Scientifique (CNRS), Unité Mixte Internationale 3199 (UMI3199), Centre for International Research in the Humanities and Social Sciences (CIRHUS), New York University, New York, New York, United States of America; 2 Service de Préhistoire de l’Université de Liège, Liège, Belgium; 3 Centre National de la Recherche Scientifique (CNRS), Unité Mixte de Recherche 5199 (UMR5199), de la Préhistoire à l'Actuel: Culture, Environnement et Anthropologie (PACEA), Université de Bordeaux, Talence, France; 4 Institute for Archaeology, History, Cultural Studies and Religion, University of Bergen, Bergen, Norway; University of Oxford, UNITED KINGDOM

## Abstract

The transition to farming is the process by which human groups switched from hunting and gathering wild resources to food production. Understanding how and to what extent the spreading of farming communities from the Near East had an impact on indigenous foraging populations in Europe has been the subject of lively debates for decades. Ethnographic and archaeological studies have shown that population replacement and admixture, trade, and long distance diffusion of cultural traits lead to detectable changes in symbolic codes expressed by associations of ornaments on the human body. Here we use personal ornaments to document changes in cultural geography during the Mesolithic-Neolithic transition. We submitted a binary matrix of 224 bead-types found at 212 European Mesolithic and 222 Early Neolithic stratigraphic units to a series of spatial and multivariate analyses. Our results reveal consistent diachronic and geographical trends in the use of personal ornaments during the Neolithisation. Adoption of novel bead-types combined with selective appropriation of old attires by incoming farmers is identified in Southern and Central Europe while cultural resistance leading to the nearly exclusive persistence of indigenous personal ornaments characterizes Northern Europe. We argue that this pattern reflects two distinct cultural trajectories with different potential for gene flow.

## Introduction

The transition to farming represents a major shift in the history of mankind. It corresponds to the process by which human groups switched from hunting and gathering wild resources to a reliance on a system of food production based on domesticated plants and animals. In the Fertile Crescent, farming, herding and sedentism progressively took place 12,000 years (y) ago, and then spread across Europe from 8,800 until 5,500 y ago [1–4]. Increasingly refined archaeological [4–6], anthropological [7–11] and chronological data (3, 10) identify a succession of profound cultural, technical and economic changes between the last Mesolithic hunter-gatherers and the first Early Neolithic farmers in Europe. Recent genetic studies reveal complex demographic events taking place over the three millennia of Neolithic spread in Europe, including multiple inputs from Neolithic migrants originating from the Near-East, but also a contribution of Mesolithic local foragers to agriculturalist societies [8,12–14]. Transition to farming was not a linear process and it was slowed down, stopped or abandoned several times in specific regions before being definitely successful all over Europe [15,16]. Its success was also concurrent with a decline in health and a raise in labor cost for food supply in many areas [7,17]. It is likely that in the past, as observed today [18], the adoption of domestication and sedentary life was promoted by new system of beliefs reflected by detectable changes in material culture [5,19,20]. Cultural items fulfilling exclusively symbolic functions are generally considered more useful than functional artifacts for detecting cultural affinities between populations and patterns of cultural change through time [21–23]. Numerous ethnographical studies have demonstrated that symbolic codes expressed by associations of ornaments on the human body change in a given area as a result of demic and cultural phenomena including population replacement and admixture, trade, and long distance diffusion of cultural traits [24–26]. Personal ornaments can therefore be considered a reliable proxy for reconstructing cultural diversity and change in past societies.

In their pioneer study, Newell et al. (1990) examined time and space diversity of personal ornaments produced by the post-glacial hunter-gatherers of Western Europe, and used ethnographic data and explicit sets of statistical analyses to identify the geography of Mesolithic social, ethnic and linguistic groups [27]. Vanhaeren and d'Errico (2006) created and explored a large scale dataset of personal ornaments present in Europe during the Early Upper Palaeolithic. By analyzing it with seriation, correspondence and interpolation methods, they identified geographically coherent clusters of sites yielding distinct ornament types and characteristic associations of types found over larger areas, and interpreted this pattern as representing the ethno-linguistic diversity of Europe during this period [28].

Here we use, for the first time, personal ornaments to comprehensively investigate cultural and population dynamics at work during transition to farming in Europe. Our analyses are aimed at documenting changes in cultural geography that occurred during the transition and identifying the mechanisms responsible for those changes in various regions of Europe.

Such reconstruction is based on the first extensive record of personal ornaments recovered from well preserved stratigraphic units, assigned to distinct archaeological cultures, and spanning over three millennia, from the Early Mesolithic to the Final Early Neolithic (S1 Dataset). We submitted this record, formatted as a binary matrix of 224 bead-types found at 212 European Mesolithic and 222 Early Neolithic stratigraphic units, to a series of spatial and multivariate analyses.

Each stratigraphic unit was assigned to one of the 48 European archaeological cultures defined in the literature according to lithic technology and ceramic production (Text A in S1 Text). Matrices of presence/absence of personal ornaments were used to calculate pairwise cultural distances between the 48 archaeological cultures using the Dice index [29]. We performed Principal Coordinates Analysis [30] and Neighbor joining [31] analyses to identify similarities between archaeological cultures. Neighbor-net analysis [32] was implemented to determine the level of borrowing and convergence in the data (Method section). The One-Way ANOSIM nonparametric procedure was used to test for significant difference between archaeological cultures [33]. The main bead types most responsible for differences between groups were identified through Similarity Percentages analysis (SIMPER) [33]. Correlation between cultural distance and geographic distance matrices was calculated using a Mantel test [34] to evaluate the effect of geographic distance on cultural diversity. Interpolation of the first axis of the Principal Coordinates Analysis (PCoA) using Spline method was used to map the Mesolithic and Early Neolithic cultural diversity [35].

Our results reveal a coherent diachronic and geographical pattern of continuity and change in the use of personal ornaments in Europe during the transition to farming. This pattern is best explained by cultural mechanisms and population dynamics rather than raw material availability or isolation-by-distance, i.e. the tendency of populations that are geographically closer to display cultural traits that are more similar than populations that are further apart [36].

Results show that i) an enduring cultural boundary exists between the North of Europe and the rest of the continent throughout the Mesolithic and the Neolithic, ii) a persistence of forager cultural attributes in Early Neolithic personal ornamentation, reflecting incorporation of Mesolithic cultural traits by emerging farming societies, is observed in the North of Europe, iii) the adoption of a large variety of novel bead types associated with a minor persistence of Mesolithic bead types characterizes Southern and Central Europe, iv) Early Neolithic geographic bead-types configuration is consistent with the two main roads of Neolithic diffusion in Europe proposed in the past on the basis of other cultural elements [4,37] and genetic data [38,39].

The history of personal attires supports the view that the Neolithisation of Central and Southern Europe took place in the context of a profound reconfiguration of symbolic and social codes while that of Northern Europe was characterized by a marked persistence of those codes. Such a persistence is consistent with a scenario of cultural continuity between the last hunters and the first farmers in the North of Europe as evidenced by other categories of the material culture [40,41]. Identified along the whole Baltic area, this pattern overarches a more diverse demographic history encompassing probable population continuity in the Eastern Baltic [42] and replacement in Scandinavia [43]. The reasons for the adoption of local symbolic traditions by incoming agriculturalists in areas where no apparent genetic exchange with autochtonous populations is identified still need to be elucidated.

Our results imply that reconstructions of the processes leading to the spread of production economies must not only consider the diffusion of novel techniques and know-how of food production but also the degree of cultural cohesiveness of local communities, which may have been more or less pervious to newcomers and their symbolic systems.

## Results

### Cultural affinities

**Fig 1:**
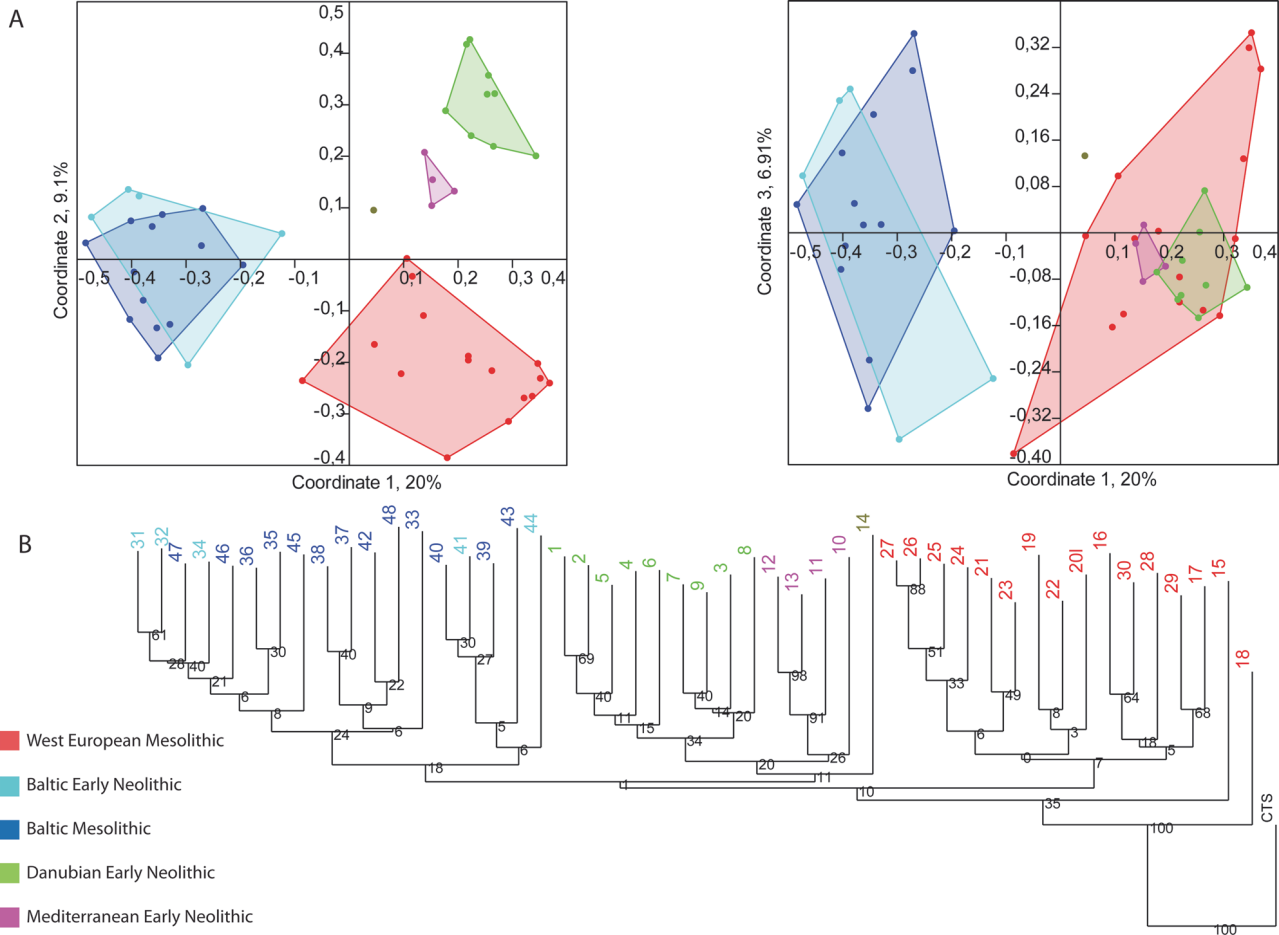
Principal Coordinates Analysis (A) and Neighbor-Joining Tree (B) of the 48 archaeological cultures using the Dice similarity index for binary data (D_*i*_). Both analyses show differentiation of the bead-type associations among the Mesolithic and Early Neolithic archaeological cultures. Archaeological cultures are color-coded according to the chronological period and European region they belong to. Numbering of archaeological cultures is detailed in Text A in S1 Text.

Fig 1A shows PCoA for the 48 archaeological cultures documented in our sample. Analysis identifies five distinct broad archaeological sets indicated with different colors, corresponding to the chronological and geographical structure of the dataset. The first PCoA axis (accounting for 20% of total variation) positions archaeological cultures from the Baltic area apart from Southern areas of Europe. The second PCoA axis (9.18%) separates South-western European Mesolithic archaeological cultures (WEM) from those attributed to the Mediterranean (MEN) and Danubian (DEN) Early Neolithic. Each of the three first axes identifies a clear overlap between Baltic Mesolithic (BM) and Baltic Early Neolithic (BEN) convex hulls, indicating a high level of bead-type sharing between the two sets. Overlap between WEM and MEN and DEN convex hulls is only visible in the PCoA3 (6.91%) reflecting minor bead-type sharing. The large convex hull of the WEM set reflects a high degree of internal heterogeneity in bead-type diversity. Smaller convex hulls for the MEN and DEN archaeological sets conversely indicate lower internal heterogeneity of bead-type diversity.

Although bootstrap values are not high for the major clusters, the Neighbor-Joining analysis identifies a geographically and chronologically coherent tree (Fig 1B). Mesolithic and Early Neolithic archaeological cultures from the Baltic area cluster together. The two West European Early Neolithic sets (DEN and MEN) appear as the closest neighbors and distinct from the Western European Mesolithic archaeological cultures. At a smaller scale, chronological structure is observed in DEN and MEN archaeological sets. The most recent Danubian Early Neolithic archaeological cultures (Linear Pottery Culture from the Paris basin [e.g. RRBP (n°5), RFBP (n°1) and Villeneuve-Saint-Germain (n°2)] are rooted on the same branch. Within the Mediterranean Early Neolithic, the tree also shows the Western recent archaeological cultures [Cardial (n°12) and Epicardial (n°13)] splitting off with Eastern earlier Impressa (n°11) and Greek Early Neolithic (n°10) archaeological cultures.

### Cultural distance

We applied the One-Way ANOSIM nonparametric procedure based on *D*_*i*_ (Table 1) to assess the significance of the five main archaeological sets identified by the PCoA (WEM, MEN, DEN, BM, BEN).

**Table 1:** ANOSIM analysis of bead-type diversity in different archaeological sets.

	WEM	BM	BEN	DEN	MEN
**WEM (R-values)**	0				
***p-values***	*0*				
**BM**	0,7487	0			
	***0*,*0001***	*0*			
**BEN**	0,7475	0,09388	0		
	***0*,*0001***	*0*,*2251*	*0*		
**DEN**	0,5837	0,8671	0,9174	0	
	***0*,*0001***	***0*,*0001***	***0*,*0005***	*0*	
**MEN**	0,4132	0,7511	0,6875	0,7731	0
	***0*,*01***	***0*,*0007***	***0*,*0085***	***0*,*0015***	*0*

Significant P-values <0.05 for pairwise comparisons are bolded for clarity (WEM: Western European Mesolithic; BEN: Baltic Early Neolithic; BM: Baltic Mesolithic; DEN: Danubian Early Neolithic; MEN: Mediterranean Early Neolithic).

Results show highly significant (R>0.75; p<0.01) and significant (R>0.5, p<0.01) bead-type differentiation between geographically (BM *vs* WEM, DEN *vs* BEN, MEN *vs* DEN) and chronologically (DEN *vs* WEM) distinct archaeological sets. Instead, a lower R value (0.25<R<0.5; p< 0.01), reflecting low cultural similarity, is observed when comparing MEN *vs* WEM. The smallest R value is observed between BEN and BM, suggesting strong similarities in the bead-types between the two sets of archaeological cultures.

This indicates that, with the single exception of the Baltic area, bead-type associations changed through time during the transition to farming, and that geographic distance between sites played a role in the cultural reshaping.

### Bead-type diversity

The SIMPER analysis (Table A in S1 Text) reveals that beads made of mammal teeth, in particular perforated red deer canines and incisors, and elk and wild boar perforated incisors were ubiquitous in the BM and the BEN sets. Shell beads were, in contrast, virtually never used at the sites composing these sets. The Baltic Early Neolithic is also characterized by the use of perforated canines of carnivores such as *Canis sp*., fox, marten, badger and seal. Amber pendants are present in these two Baltic archaeological sets exclusively. Differences in the bead-types between the WEM, the DEN, and the MEN are mostly reflected by the absence of shaped ornaments made of shell and stone in the first of these sets. Shells and mammal teeth bead-types are highly diversified in the WEM with *Dentalium* shells, perforated *Columbella rustica*, *Nassarius sp*., and perforated or notched red deer canines being the more commonly used bead-types. MEN also differs from DEN in that it presents a lower diversity of shaped ornaments made of shells and stones.

Long *Spondylus* tubular beads, triangular shells beads and *Unio sp*. beads only occur in DEN archaeological cultures. Perforated shell and teeth are a background common to WEM, DEN and MEN.

### Network analysis

**Fig 2:**
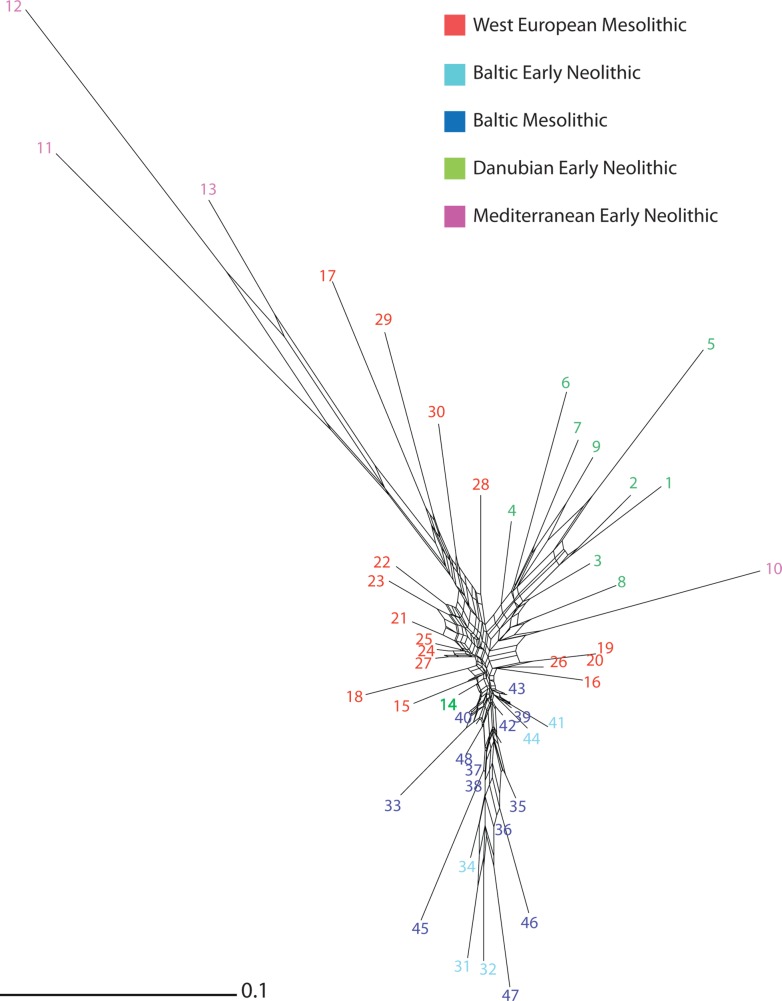
Neighbor-Net tree of the 48 Mesolithic and Early Neolithic cultures (Text A in S1 Text), using the D_*i*_. Reticulations represent evidence of borrowing or exchange and are visible within and among each archaeological culture. Archaeological cultures are color-coded according to the chronological period and European region they belong to.

The NeighborNet analysis (Fig 2) shows sharp separation of three main sets of archaeological cultures, comprising the Baltic archaeological cultures, DEN and MEN, except the Greek Early Neolithic (n°10) that does not cluster with any other MEN archaeological cultures. Although the sets are clearly discernible, the overlapping boxes point to conflicting splits in the data [44]. This is especially clear in the WEM clade, which exhibits a highly reticulated structure. The MEN set divides into two relatively well defined branches, one comprising Cardial (n°12) and Epicardial (n°13) archaeological cultures, the other Impressa (n°11). The DEN set follows a similar tendency with its most recent archaeological cultures [VSG (n°2), RRBP (n°5), RFBP (n°1)] appearing as closely related. This analysis indentifies the same chronological and geographical trends as those determined by the PCoA, as well as filiations between cultural facies. The fair amount of reticulations demonstrates the degree of relatedness between the different archaeological cultures of this period (Fig 1C). Estimates of the overall tree-likeness/boxiness of the network yielded an average delta score of 0.3 and Q-residual score of 0.02.

### Geographic pattern and spatial correlation

The mantel test shows a low albeit significant correlation (R = 0.23085, p = 0.000001) between geographic and cultural distance between sites indicating that geography explains a small portion of the cultural variance. Pairs of spatially close sites will be more culturally similar than distant sites.

**Fig 3:**
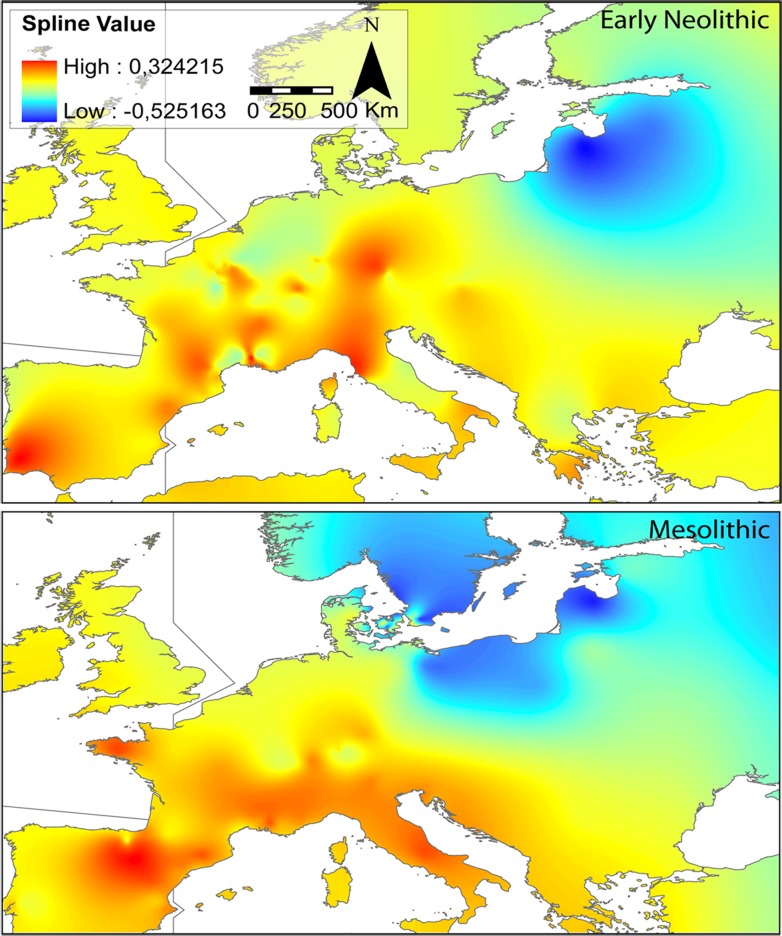
Spline interpolation of the first axis of the Principal Coordinates Analysis. Geographic structure differentiation between Mesolithic and Early Neolithic indicates reshaping of the bead-type diversity in Europe during the transition to farming. Maps were made by S.R. by using ArcGIS 9.3.1 software.

The map of interpolated bead-type diversities throughout Europe, based on 48 archaeological cultures, shows a persistent sharp divide between bead-type associations from the Baltic area and those from Southern Europe during the Mesolithic and the beginning of the Neolithic (Fig 3). The highest interpolated values (in red) are distributed on the whole of Southern Europe during the Mesolithic. At the beginning of the Neolithic, the highest values are mainly restricted to Portugal, Northern Italy and Eastern Central Europe.

## Discussion and Conclusion

Analysis of Mesolithic and Neolithic bead-type associations throughout Europe identified diachronic and geographical patterns in the use of personal ornaments. Our main findings can be summarized as follows: (1) bead-type diversity decreases northwards, (2) well-defined long-lasting geographic boundaries persist through time between the South and the North of Europe, (3) bead-type diversity and geographic distances are only slightly correlated; (4) the Mesolithic and Early Neolithic of Northern Europe show a clear continuity in bead-types while (5) a significant discontinuity is observed in Central and Southern Europe, (6) bead-type associations at the longitudinal extremes (Iberia and Italy on the one hand and Eastern and Central Europe on the other hand) are more similar than expected considering their geographic location.

Raw material availability does not account for the observed pattern. Long distance trade of objects used as beads, well attested during the Mesolithic and the Early Neolithic [45–49], supplied the raw materials necessary to make beads in regions where they were desired but naturally rare or absent. Absence of amber ornaments outside the Baltic area cannot be attributed to the lack of this raw material. Amber outcrops are documented in many regions of Europe [50–52] and were exploited during the Upper Paleolithic [53,54], and probably the Bronze Age [51]. Raw material availability also fails to explain the near complete absence of perforated shells in the Baltic area, where numerous suitable shell species were available, at least at the beginning of the so called *Littorina* transgression (circa 8000–7200 cal BP, [55–57]. It also does not explain the substantial change in bead type associations and reshaping of bead diversity observed in the Early Neolithic of Central and Southern Europe, where no change in raw material accessibility occurred between the Mesolithic and the Neolithic."

Since raw material availability and mechanisms of isolation-by-distance are not the determining factors behind the reconstructed trend we must conclude that such a trend reflects cultural processes.

With the Early Neolithic, a substantial turn-over in bead-type associations is observed in the Mediterranean Area, Central and South-Eastern Europe. This occurs in conjunction with the construction of similar bead-type associations around the Mediterranean area of Europe on the one hand, and in the Balkans and Central Europe on the other hand, which are incompatible with mechanisms of isolation-by-distance. This change likely reflects the Neolithic spread from Anatolia and the Near East through the Bosphorus and the Mediterranean islands to the West of Europe [38,58,59].

**Fig 4:**
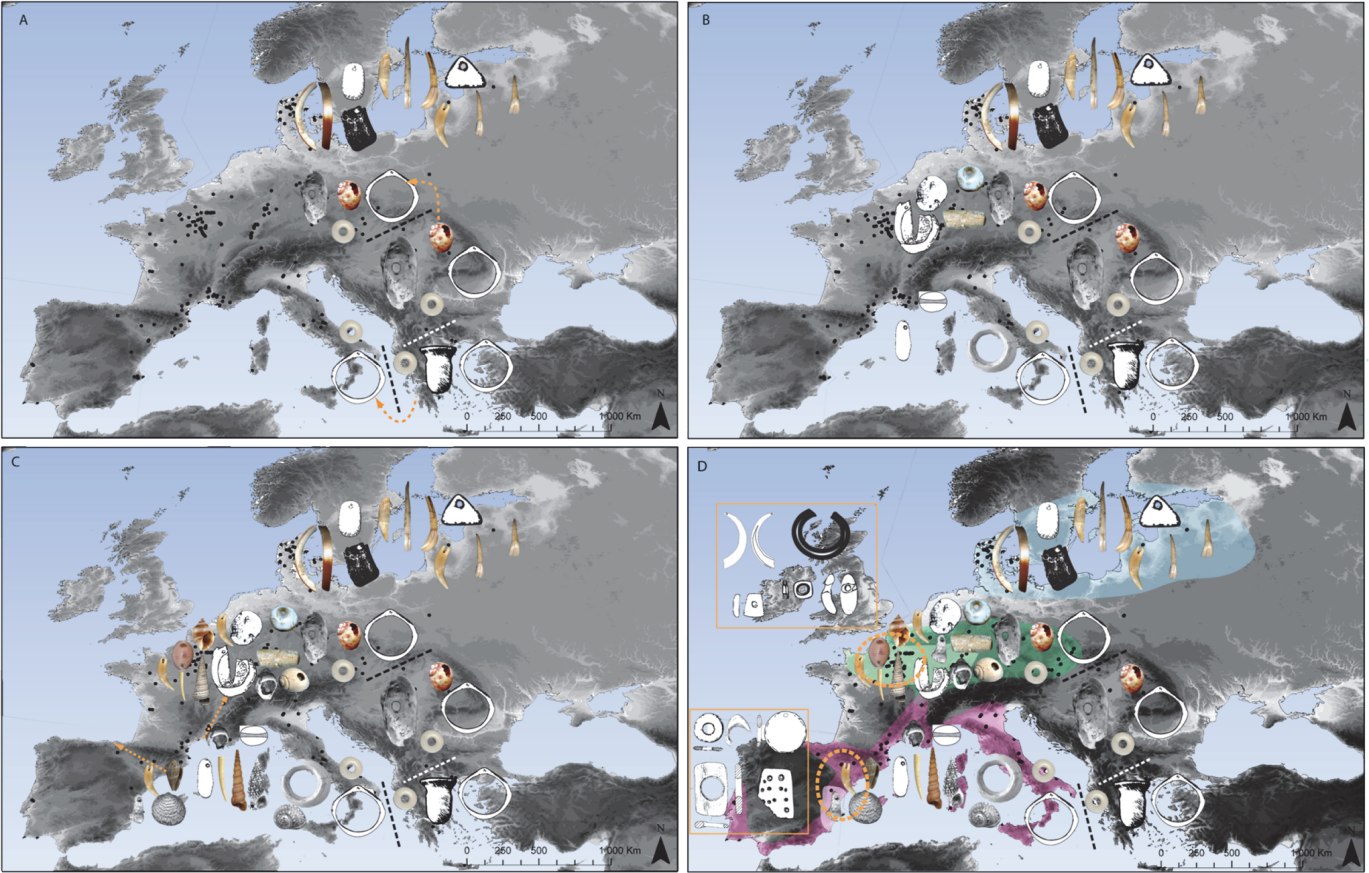
Cartography of the Early Neolithic bead-type configuration. A) Large-scale diffusion of exclusively Neolithic ornament types; B) Diversification of Early Neolithic ornament types; C) Persistence of Mesolithic bead-types in the Neolithic; D) Emergences of new bead types at regional scale. Dotted black lines indicate the major shifts in bead-type associations in South East Europe. Dotted orange ellipses show the two areas where numerous new bead-types were adopted during the end of the Early Neolithic. Color shaded areas indicate the geographic distribution of the MEN (pink), DEN (green) and BEN (blue) archaeological cultures considered in the analysis. Maps were made by S.R. by using ArcGIS 9.3.1 software. Some bead types were redrawn from [64–67].

Results also show a limited but significant persistence of Mesolithic bead-types in Early Neolithic personal ornamentation (Fig 4C). This clearly indicates that cultural traits and presumably also individuals circulated from one society to another. Genetic [60,61] and isotopic data [62,63] are consistent with our results. They identify complex demic processes, comprising local forager alongside with Neolithic migrant contributions to the European gene pool and both incorporation of Neolithic migrants into forager communities and conversely, incorporation of foragers into Neolithic communities. Appropriation and incorporation of cultural traits may have facilitated the circulation of individuals from one community to another and led to the persistence of forager cultural attributes. This process might have represented a successful strategy for farmers seeking to spread in territories where strong foraging communities were implanted.

Mesolithic and Early Neolithic bead-type associations from Northern areas are remarkably homogeneous; those from Southern areas more variable. During the Mesolithic, the Mediterranean area, Atlantic Coast and Eastern Central Europe, display the highest values of bead-type diversity. Presence in the Early Neolithic of these regions of bead types already used during the Mesolithic leads to the preservation of high levels of variation, especially in Portugal, Northern Italy and Eastern Central Europe. This result shows that the Mesolithic cultures from Southern Europe had important influence on the amount and distribution of cultural variation detected among Southern and Central European Early Neolithic cultures.

The Early Neolithic in Western Europe is also characterized by the appearance of new bead-types, unknown in the local Mesolithic as in the Near East Early Neolithic [64,65]. These new types demonstrate that spreading farming societies progressively developed new cultural traits concurrent with increasing distance from the Core Area (Fig 4).

Bead associations from Northern Europe tell us a different story. They reveal a nearly complete continuity in bead-types between the Mesolithic and the Neolithic, and no adoption of the Neolithic bead-types identified in the rest of the continent (Fig 1A, Fig 1C). This result is in agreement with trends evidenced by other categories of the material culture [40,41]. In the Eastern Baltic, continuity in bead types is associated with population continuity [42]. This suggests that Neolithic production techniques were adopted by local populations who preserved their ornaments. In Scandinavia, genetic data support a scenario of population replacement [14,43]. The higher diversity of bead types observed at the beginning of the Neolithic in this region may reflect a situation in which incoming farmers adopted ornament types used by the local Mesolithic while, at the same time, enlarging their panoply and changing the way in which ornaments were integrated in their attires.

No significant changes in bead types, apart from an increased use of perforated mammal teeth and more diverse shaped beads made of shell and stone [68,69], are observed between the Early and the Middle Neolithic of Central Europe, which chronologically overlaps the Baltic Early Neolithic. This indicates that differences in bead types between Central and North of Europe remained at work throughout the Early and the Middle Neolithic and represented during this time span an enduring cultural divide between the two areas. Our results show that the spread of the Neolithic resulted in two remarkably different cultural histories between the Central and the South Europe on the one hand, and the North of Europe on the other hand. In these two areas Early Neolithic farmers faced different challenges, implying dissimilar opportunities for cultural exchange and demic expansion. Changes in personal ornamentation identified at a regional scale show that population dynamics are not only ruled by the diffusion of new subsistence strategies and related technologies, but also by the renewal of symbolic standards linked to social norms and systems of belief. The transition to farming emerges as a complex process in which techniques, know-how, symbols and systems of belief are not transferred as a package. Techniques developed by farming societies largely spread throughout Europe through demic diffusion or adoption by foraging communities. Symbolic items show in some instances a high degree of resilience and may have been incorporated in the symbolic codes of spreading farming communities. Identifying to what extent, in each region of Europe, genes and cultural traits moved from a population to another during this period of transition is now within our grasp and can be achieved by contrasting our dataset with the biological data available for the human populations living in Europe during this period [11,12,70,71]. Such a comparison may lead to the understanding of the mechanisms and historical processes that have largely contributed to the shaping of present day European cultural and biological population diversity.

## Methods

### Sample

A geospatial database of bead-types found at European Mesolithic and Early Neolithic sites was created by using ArcView GIS software. Data were obtained from the literature, and the direct analysis of published and unpublished archaeological collections (S1 Dataset).

The typology of Mesolithic and Early Neolithic personal ornaments takes into account cross-cultural studies on the classification of beads and criteria used to classify archaeological artefacts [72,73]. Discrete bead-types were created with reference to raw material, morphology, system of suspension (e.g., perforation, groove), size, section and profile (Text B, Figures A-C and Tables B-F in S1 Text). In the case of animal teeth we also considered anatomical and species identification.

Taxonomic identifications given in the literature for marine and fresh water shells were updated and standardized using CLEMAM, Check List of European Marine Mollusca Database (http://www.somali.asso.fr/clemam/index.clemam.html, date of access: 05/11/2013), CLECOM, Check List of European Continental Mollusca (http://www.gnm.se/gnm/clecom/clecom.asp?res=1024, date of access: 12/10/2012) and the Paleobiology database for the fossil species (http://www.paleodb.org/cgi-bin/bridge.pl, date of access:20/12/2013).

Shell bead types refer to species when the latter can be visually discriminated; otherwise they refer to genus. Taphonomic studies [74,75] indicate that not all objects with a perforation or a gouge are personal ornaments. We have excluded from our database objects bearing no compelling traces of human modification and, in general, included objects small enough to be worn as personal ornaments, and showing clear anthropogenic suspension devices.

e retained sample includes 1009 occurrences of 224 mutually exclusive bead-types found at 434 archaeological layers from Europe. These sites are attributed, according to lithic technology and ceramic productions, to 48 distinct archaeological cultures (Text A in S1 Text).

### Datasets

The amount and type of ornaments recovered at an archaeological site depend on the site function (ex. domestic, funeral) and the excavation methods. Systematic sieving of the sediment with small mesh grids can for example significantly increase the number of small beads recovered [76]. Since due to the above reasons proportions of ornaments recovered at archaeological sites cannot be considered representative of the importance attributed to specific ornament types by Mesolithic and Early Neolithic populations, in our analyses we only used presence/ absence of data.

Presence or absence of bead-types was coded as ‘1’ or ‘0’ respectively, to produce a matrix of 437 archaeological sites coded across 223 binary traits. Binary variables were rejected if bead-types were present at less than three sites.

The presence/absence matrix was converted to a Dice distance matrix reflecting pairwise cultural distances between sites and archaeological cultures. The Dice distance is particularly appropriate for analyzing this cultural dataset because it stresses joint occurrences more than mismatches [29]. The Dice distance for each pair of archaeological sites was calculated as the number of bead-types that are present in the two sites, divided by the sum of the number of bead-types that are present in one or both of the sites.

$$Di=\frac{2Nab}{\left[2Nab+Na+Nb\right]}$$

*Na*: Number of bead-types present in site a

*Nb*: Number of bead-types present in site b

*Nab*: Common bead-types between sites a and b

The parameter *D*_*i*_ is the proportion of total variability owing to differences between sites, and was calculated pairwise as a measure of bead-type divergence between sites.

Geographic coordinates were used to calculate pairwise Euclidian geographical distance between archaeological sites.

### Data processing and analysis

Pairwise *D*_*i*_ was entered into a Principal Coordinates Analysis (PCoA) performed in PAST software, [77] to identify and plot similarities in terms of bead-type diversity between sites. This technique was chosen over other ordination techniques because of PCoA's ability to utilize binary data [30]. Mapping of the PCoA was performed by using Spline interpolation [35] run through ArcGIS 9.3.1 software.

Pairewise *Di* was also used in a neighbor-joining tree [31] with the bead-types association from the Early Upper Paleolithic site of Les Cottés (38 kyr) as an outgroup [78]. Interior-branch reliability for the tree was tested by means of 1000 bootstrap replications using PAST software [77].

The One-Way ANOSIM nonparametric procedure based on *D*_*i*_ was used to test for significant difference between archaeological cultures and sets [33], and run through PAST with 10000 permutations. ANOSIM shows that pairwise groups with R-values >0.75 are well separated; groups with R>0.5 are partially overlapping but noticeably different and groups with R<0.25 are scarcely separable.

In addition, Similarity Percentages analysis [SIMPER, [33]] based on Bray-Curtis distances was used to identify the bead types most responsible for differences between archaeological sets.

Pairwise *D*_*i*_ was also used in a ‘Neighbor-net’ analysis [32] to determine conflicts within the data represented by reticulations or joining among branches. From the shape of the Neighbor-Net network, we can infer the level of borrowing and convergence that occurred between the different archaeological cultures. The analysis was performed in SPLITSTREE 4 using standard settings [32].

The isolation-by-geographic distance model predicts a positive relationship between increased cultural differentiation and geographic distance. To account for the effect of geographic distance on cultural diversity, correlation between cultural distance and geographic distance matrices was calculated using a Mantel test [34], performed in PASSaGE 2 [79].

## Supporting Information

S1 Dataset Database of the archaeological sites and layers used in the analysis. (XLS)

S1 Text Method and criteria used for the design of the bead-type database. (PDF)
