# Supplementary material for: Ornaments Reveal Resistance of North European Cultures to the Spread of Farming
Source: PLoS One. 2015 Apr 8;10(4):e0121166. doi: 10.1371/journal.pone.0121166 (PMC4390204; doi:10.1371/journal.pone.0121166)
Supplement: S1 Text — (PDF) [file pone.0121166.s002.pdf]

Ornaments reveal resistance of North European cultures to the spread of farming

Solange Rigaud, Francesco d'Errico, Marian Vanhaeren

SUPPORTING INFORMATION (SI)

SUPPORTING TEXTS 2

TEXT A: Archaeological cultures plotted in figures 1 and 2 2

TEXT B: Bead typology used to create the binary data cultural matrix 4

SUPPORTING FIGURES 5

Fig. A. Examples of the shells species used as beads and recorded in the database 5

Fig. B. Examples of vertebrae teeth used as beads and recorded in the database 6

Fig. C. Classification of beads, pendants, platelets and bracelets. Some bead types were redrawn from [34,35,38,39]. 7

SUPPORTING TABLES 8

Table A. SIMPER analysis identifying bead-types most responsible for cultural differences between Mesolithic and Early Neolithic archaeological datasets. Overall average dissimilarity : 91,11 8

Table B. List of the shell taxa used as beads and recorded in the database 12

Table C. List of the vertebrate bones and teeth recorded in the database (I: incisor, C: canine, M: molar, indet: indeterminate) 13

Table D. List of the mineral raw materials recorded in the database 14

Table E. List of the bead-types recorded in the database 14

Table F. List of the bead-types present in each of the archaeological cultures 18

SUPPORTING DATASET 19

DATASET S1 (Separate Excel sheet). Database of the Mesolithic and Early Neolithic European sites used in the analysis. 19

REFERENCES FOR SUPPORTING INFORMATION 19

SUPPORTING TEXTS

TEXT A: Archaeological cultures plotted in figures 1 and 2

1: Rubané Final du Bassin Parisien, Early Neolithic, Paris Basin, 5000-4800 cal BC[1].

2: Villeneuve Saint-Germain, Early Neolithic, Paris Basin, Brittany and Belgium, 4950–4650 cal BC[2].

3: Middle Linearbandkeramik, Early Neolithic, Central Europe, circa 5200 cal BC[3].

4: Late Linearbandkeramik, Early Neolithic, Central Europe, circa 5100 cal BC[3].

5: Rubané Récent du Bassin Parisien, Early Neolithic, Paris basin, 5100-4900 cal BC[4].

6: Hinkelstein, Early Neolithic, Middle Rhine valley, 5500-500 cal BC[5].

7: Linearbandkeramik sites from central Europe that were directly or indirectly attributed to the Early Neolithic with no further precision in the literature, 5500-5000 cal BC.

8: Early Linearbandkeramik, Early Neolithic, Central Europe, 5600-5450 cal BC[3].

9: Final Linearbandkeramik, Early Neolithic, Central Europe, circa 5000 cal BC[3].

10: Early Neolithic archaeological sites located in Crete and Greece, 6800-6600 cal BC[6].

11: Impressa, Early Neolithic, Adriatic coast, Italy and East of the lower Rhone valley, 5700-5600 cal BC[7].

12: Cardial, Early Neolithic, Western Mediterranean, 5600-4800 cal BC[8].

13: Epicardial, Early Neolithic, Western Mediterranean from Western Alps and Lower Rhône valley to Andalusia, 5400-4500 cal BC[7].

14: Starcevo, Early Neolithic, Danubian plain, Morava valley, Serbia and northwest Bulgaria, 6300-5500 cal BC [9].

15: Late Mesolithic sites from Lower Danube Valley, 7500-6400 cal BC[10,11].

16: Obanian, Late Mesolithic and Early Neolithic shell middens from Western Scotland, 7500–3900 cal BC[12].

17: Early and Middle Mesolithic, Southwest Europe, 8500- 7000 cal BC[13].

18: Beuronian, Mesolithic, Germany, 8500-6500 cal BC[14].

19: Asturian, Late Mesolithic shell middens from North Iberia Atlantic coast, 8100-5500 cal BC[15].

20: Early Mesolithic from Britain, 8600-7800 cal BC[16].

21: Early Mesolithic from the Lower Danube Valley, 8400-7500 cal BC[10,11].

22: Mesolitico Geometrico, Late Mesolithic from the Iberian Peninsula, 7000-5200 cal BC[17,18].

23: Mesolithico muescas y denticulados, Early Mesolithic from the Iberian Peninsula, 8200-6400 cal BC[19].

24: Mesolithico Macrolitico, Early Mesolithic from the Iberian Peninsula, 9800-7400 cal BC[20].

25: Cocina, Late Mesolithic from the Iberian Peninsula, 6500-5500 cal BC[17].

26: Late Mesolithic layers from Franchti Cave (Greece), 7050-6300 cal BC[21].

27: Early Mesolithic from Franchti cave (Greece), 8750-8200 cal BC[21].

28: Personal ornaments from the Middle Mesolithic site of la Chaussée-Tirancourt (France) dated circa to 7600 cal BC[22].

29: Castelnovian, Late Mesolithic, Southwest Europe, 6500-5000 cal BC[23].

30: Teviecian, North Atlantic coast Late Mesolithic culture, Brittany, 5700-5000 cal BC[24].

31: Early Neolithic from the Baltic region, including archaeological layers from sites located in Scandinavia and Eastern coastal Baltic areas (Lithuania, Latvia, Estonia, North of Poland). Archaeological layers are directly or indirectly attributed to the Early Neolithic with no further precision in the literature (5500-4200 cal BC).

32: Burials from Zvejnieki cemetery (Latvia) where both Mesolithic and Early Neolithic features are identified[25].

33: Early Mesolithic archaeological layers from sites located in Scandinavia and Eastern coastal Baltic areas (Lithuania, Latvia, Estonia, North of Poland). Archaeological layers are directly or indirectly attributed to the Early Mesolithic with no further precision (9500-6500 cal BC).

34: Narva culture, Early Neolithic, North East Poland, Lithuania, Latvia, Estonia and western Russia, 5500-4200 cal BC[26].

35: Middle Mesolithic archaeological layers from sites located in Scandinavia and Eastern coastal Baltic areas (Lithuania, Latvia, Estonia, North of Poland). Archaeological layers are directly or indirectly attributed to the Middle Mesolithic with no further precision (5500-5200 cal BC).

36: Early Ertebølle, beginning of the Late Mesolithic, Southern Scandinavia and Northern Germany, 5400-4800 cal BC[27,28].

37: Late Kongemosian, final Middle Mesolithic, South Scandinavia and Denmark, 5700-5200 cal BC[27].

38: Kongemosian, Middle Mesolithic, South Scandinavia and Denmark, 6500-5400 cal BC[27,28].

39: Ertebølle, Late Mesolithic, Southern Scandinavia and Northern Germany, 5400-3800 cal BC[27].

40: Mid-Ertebølle, Late Mesolithic, Southern Scandinavia and Northern Germany, 4800-4300 cal BC[27].

41: Funnel Beaker culture, Early Neolithic, North of Central Europe and Southern Scandinavia, 4200-2800 cal BC[29].

42: Janislawice, Late Mesolithic, Poland and Lithuania, 6400-5500 cal BC[30].

43: Komornica, Early Mesolithic, Northeast Poland, 8500-7000 cal BC[31].

44: Early Neolithic archaeological layers from Karelia and Volga, 6500 -5400 cal BC[32].

45: Kunda, Mesolithic, Northeast Poland, Lithuania, Latvia, Estonia and western Russia, 8500-5500 cal BC[26].

46: Late Mesolithic from archaeological layers of sites located in Scandinavia and Eastern coastal Baltic areas (Lithuania, Latvia, Estonia, North of Poland). Archaeological layers are directly or indirectly attributed to the Late Mesolithic with no further precision (5500-4200).

47: Maglemosian, Early Mesolithic, South Scandinavia and Denmark, 9600-6500 cal BC[33].

48: Mesolithic from Karelia and Volga, 9500-6500 cal BC[32].

TEXT B: Bead typology used to create the binary data cultural matrix

We have simplified and homogenized bead typologies previously published [34–42] in order to create mutually exclusive bead-types.

Bead-types included in the database are characterized by their shape, raw material and system of suspension. Unshaped objects include small size Bivalvias, gastropods, scaphopods, mammal's teeth and short bones simply perforated, grooved or incised for suspension. Distinction is made between beads, pendants, platelets and bracelets.

Beads are made of 78 species of modern marine and 26 fossil shells, 9 freshwater shells, 2 terrestrial snails (Table S1, Fig. S1), the teeth or bones from 25 different species of vertebrates (Table S2, Fig. S2), amber, and 13 different types of mineral resources (Table S3), including 8 petrographic sub-types.

Shaped bead-types with common, or very similar, characteristics in terms of shape, section and profile were grouped into eleven major classes: discoid, oval, square, rectangular, trapezoidal and triangular beads, beads drop-shaped with a distal enlargement, round beads, bi-conical beads, tubular beads with circular section and linear profile, tubular beads with circular section and curved profile (Fig. S3; 1-11).

Pendants and platelets are described according to their width, elongation and number of perforations. Four types of pendants: arciform, straight, triangular and trapezoidal (Fig. S3; 12-20) and two categories of platelets (Fig. S3; 18-20): round to oval and square to rectangular, were considered.

Bracelets were ordered into four distinct types defined according to their section: oval (Fig. S3; 21-23), triangular (Fig. S3; 26-28), flat rectangular (Fig. S3; 29) and grooved ellipsoidal (Fig. S3; 24, 25).

Ear Studs, and antler and bone rings are additional bead types included in the database.

SUPPORTING FIGURES

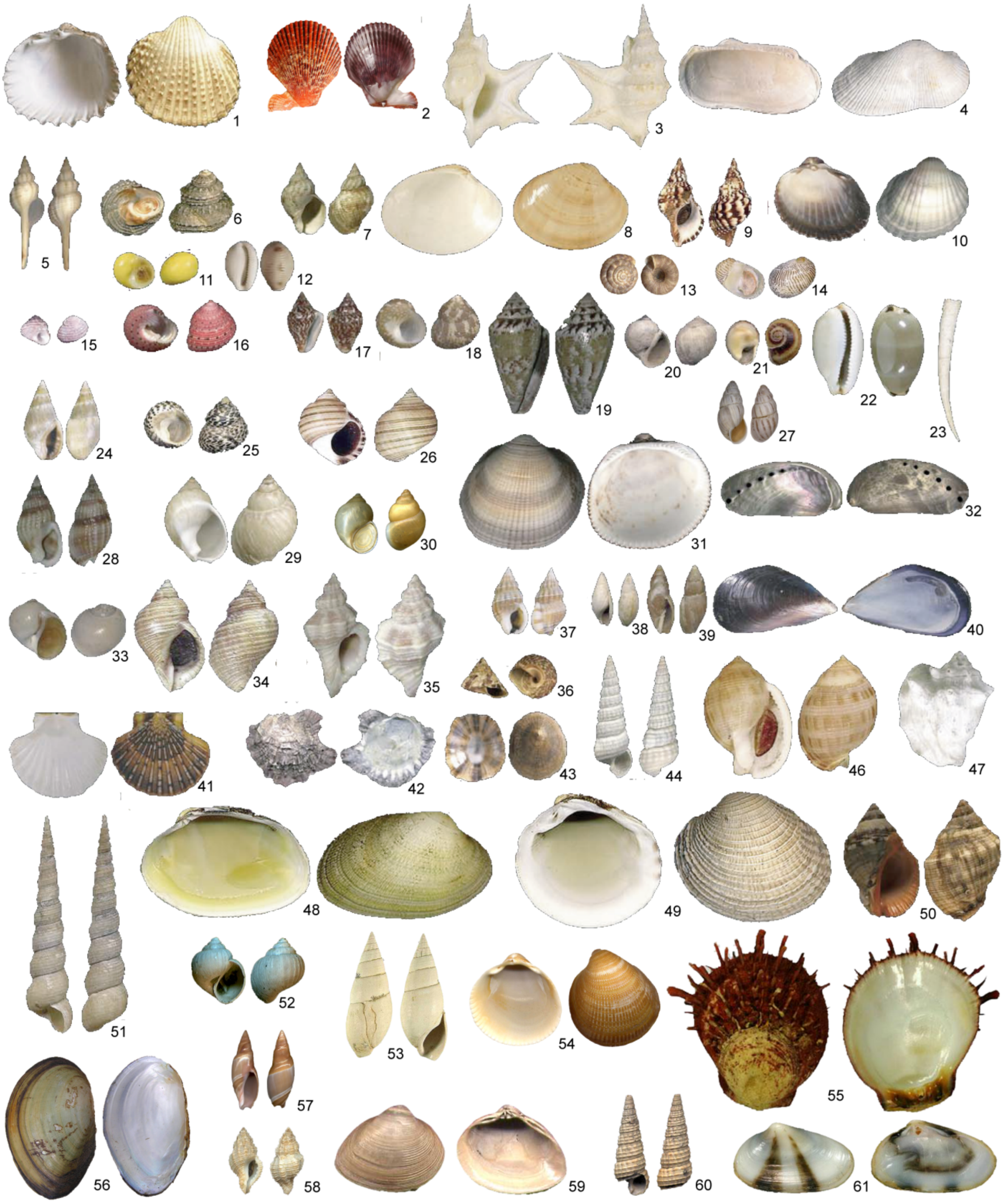

1) *Acanthocardia* sp., 2) *Chlamys varia*, 3) *Aporrhais pespelecani*, 4) *Arca noae*, 5) *Fusus* sp., 6) *Architectonatica* sp., 7) *Buccinum* sp., 8) *Callista chione*, 9) *Charonia lampas*, 10) *Cerastoderma edule*, 11) *Littorina obtusata*, 12) *Trivia monacha*, 13) *Discus rotundatus*, 14) *Theodoxus* sp., 15) *Homalopoma sanguineum*, 16) *Clanculus* sp., 17) *Columbella rustica*, 18) *Gibbula* sp., 19) *Conus* sp., 20, *Crommium* sp., 21) *Cyclope neritea*, 22) *Luria lurida*, 23) *Antalis* sp., 24) *Nassarius corniculum*, 25) *Osilinus* sp., 26) *Littorina littorea*, 27) *Zebrina detrita*, 28) *Nassarius reticulatus*, 29) *Nassarius mutabilis*, 30) *Bythinia tentaculata*, 31) *Glycymeris* sp., 32) *Haliotis* sp., 33) *Natica* sp., 34) *Nucella lapillus*, 35) *Ocenebra* sp. 36) *Trochus* sp., 37) *Nassarius incrassatus*, 38) *Melanopsis* sp., 39) *Mitra* sp., 40) *Mytilus* sp., 41) *Pecten* sp., 42) *Ostrea* sp. 43) *Patella* sp., 44) *Potamides* sp., 46) *Phalium saburon*, 47) *Strombus* sp., 48) *Tapes* sp., 49) *Venus* sp., 50) *Stramonita haemastoma*, 51) *Turitella* sp., 52) *Ampullina* sp., 53) *Bayania* sp., 54) *Laevicardium* sp., 55) *Spondylus gaederopus*, 56) *Unio* sp., 57) *Amalda* sp., 58) *Trophon* sp., 59) *Corbicula* sp., 60) *Tympanotonos* sp., 61) *Donax* sp.

Fig. A. Examples of the shells species used as beads and recorded in the database

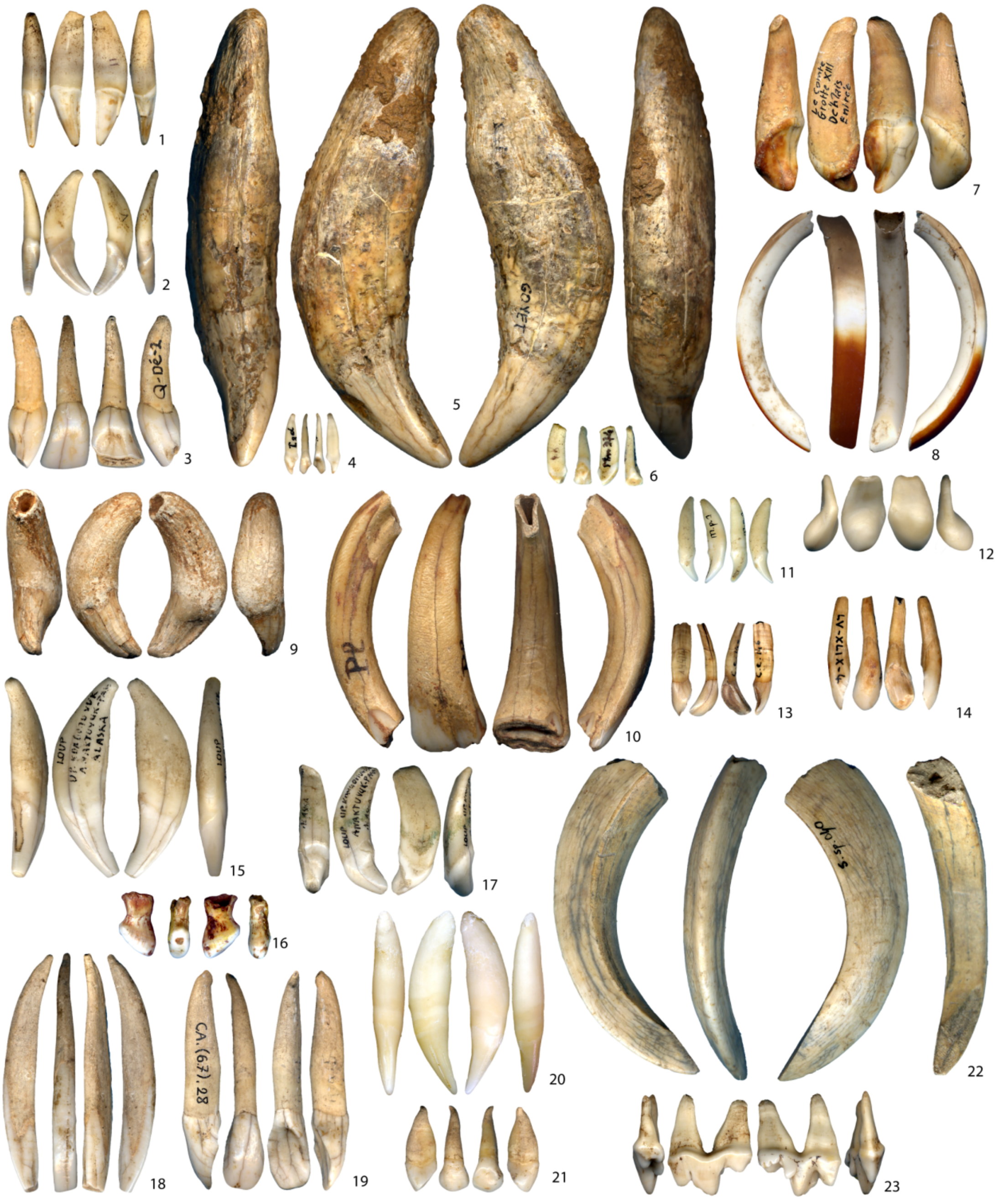

1) Badger canine, 2) Fox canine, 3) Bovidae incisor, 4) Fox incisor, 5) Bear canine, 6) Badger incisor, 7) Bear incisor, 8) Beaver incisor, 9) Horse canine, 10) Horse incisor, 11) Polecate canine, 12) Red deer canine, 13) Red deer incisor, 14) Ibex incisor, 15) Canidae canine, 16) Rutilus sp. pharyngeal tooth, 17) Canidae incisor, 18) Wild boar incisor, 19) Elk incisor, 20) Lynx canine, 21) Human incisor, 22) Wild boar canine, 23) Canidae molar.

Fig. B. Examples of vertebrae teeth used as beads and recorded in the database

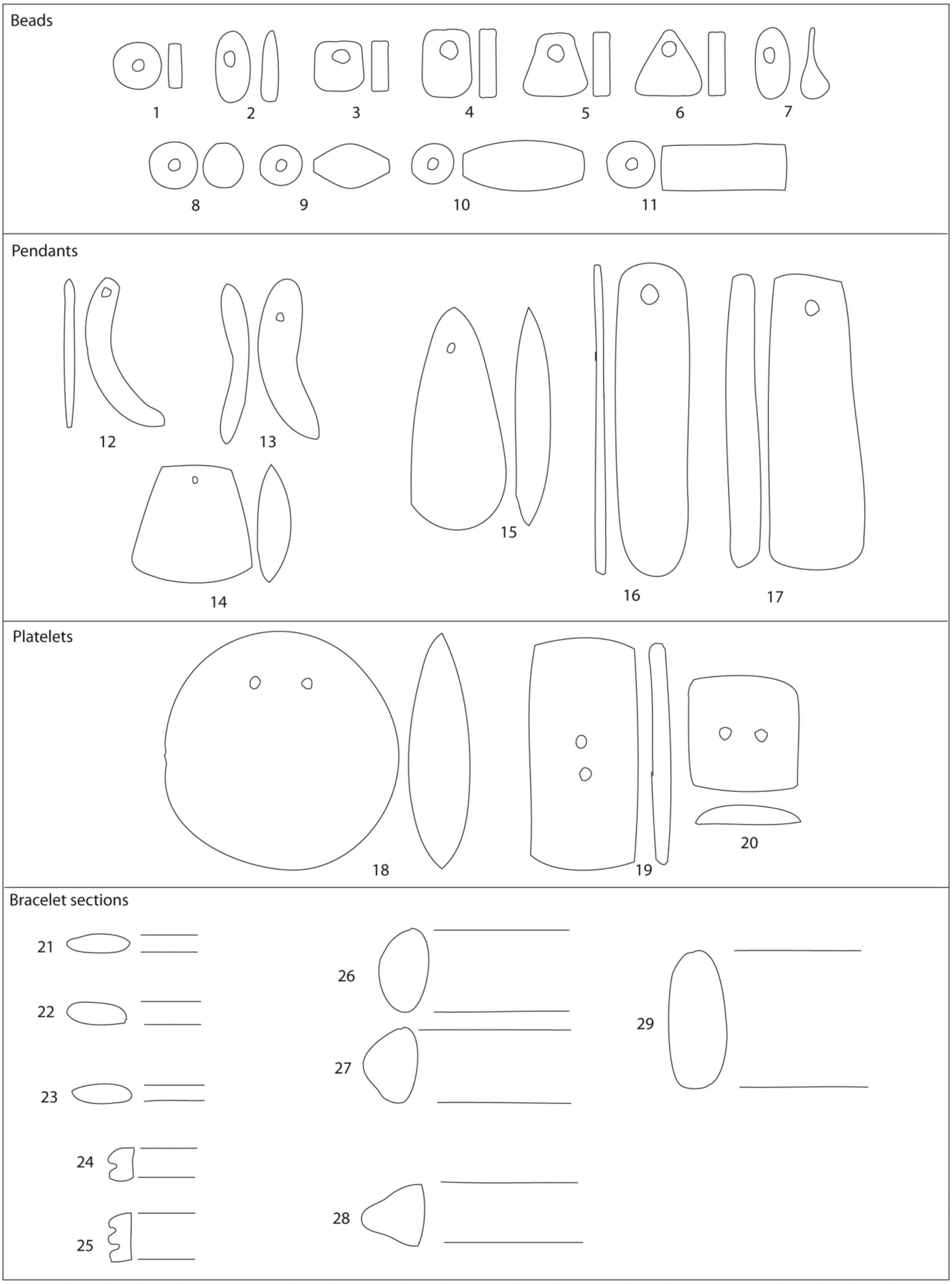

Fig. C. Classification of beads, pendants, platelets and bracelets. Some bead types were redrawn from [34,35,38,39].

SUPPORTING TABLES

Table A. SIMPER analysis identifying bead-types most responsible for cultural differences between Mesolithic and Early Neolithic archaeological datasets. Overall average dissimilarity : 91,11

Taxon	Av. dissim	Contrib. %	Cumulative %	WEM	BM	BEN	DEN	MEN
**Antalis**	2.432	2.67	2.67	0.625	0	0	1	1
**ICelP**	2.191	2.405	5.074	0.125	0.692	0.8	0	0.25
**IAlcP**	2.177	2.39	7.464	0	0.769	0.6	0	0
**CCelP**	2.104	2.31	9.773	0.563	0.538	0.2	0.444	0.75
**Columbel**	2.012	2.208	11.98	0.625	0	0	0.222	0.75
**ISusP**	1.869	2.051	14.03	0	0.615	0.6	0	0.25
**Gly**	1.63	1.789	15.82	0.438	0	0	0.333	0.75
**nassariu**	1.611	1.768	17.59	0.625	0	0	0	0.5
**CSusP**	1.513	1.661	19.25	0.0625	0.308	0.4	0.556	0.75
**PSheDsc**	1.445	1.586	20.84	0	0	0	0.889	1
**PtOs**	1.418	1.557	22.39	0.25	0.308	0.2	0.111	0.75
**Cyclope**	1.404	1.541	23.93	0.438	0	0	0.111	0.5
**IBosP**	1.385	1.52	25.45	0	0.462	0.6	0	0.25
**Trivia**	1.352	1.484	26.94	0.438	0	0	0.222	0.75
**Pecten**	1.35	1.482	28.42	0.438	0	0	0.111	0.75
**Spond**	1.279	1.404	29.82	0.0625	0	0	0.778	0.5
**PSheOv**	1.252	1.374	31.2	0	0	0	0.778	0.75
**Theodoxu**	1.244	1.366	32.56	0.313	0.154	0	0.333	0.5
**PCalDsc**	1.233	1.354	33.92	0.0625	0	0	0.667	0.75
**PtAmb**	1.215	1.333	35.25	0.0625	0.0769	0.6	0	0
**POsTub**	1.202	1.319	36.57	0.125	0.154	0.2	0.222	1
**CCanP**	1.161	1.274	37.84	0.0625	0.308	0.6	0.222	0.5
**BrcSchi**	1.147	1.259	39.1	0	0	0.2	0.556	0.5
**Littor**	1.121	1.23	40.33	0.438	0	0	0.111	0
**Cerith**	1.101	1.209	41.54	0.375	0	0	0.111	0.75
**PAmb**	1.079	1.184	42.73	0	0.308	0.4	0	0
**PSpoTub**	1.061	1.165	43.89	0	0	0	0.778	0
**CVulP**	1.049	1.151	45.04	0.188	0.0769	0.6	0.111	0.75
**AnOs**	1.043	1.145	46.19	0	0	0	0.667	0.5
**Natica**	1.011	1.109	47.3	0.25	0	0	0.333	0
**SpoInc**	0.9627	1.057	48.35	0	0	0	0.667	0.25
**Cerast**	0.9612	1.055	49.41	0.375	0	0	0.111	0.75
**ICastP**	0.9471	1.039	50.45	0	0.308	0.4	0	0
**Nucella**	0.9389	1.031	51.48	0.188	0	0	0.444	0
**PtGal**	0.9162	1.006	52.48	0.25	0.0769	0	0.222	0.25
**CUrsP**	0.8286	0.9094	53.39	0	0.308	0.4	0	0.25
**ICanP**	0.8206	0.9007	54.29	0	0.308	0.4	0	0
**Lithogly**	0.7581	0.8321	55.13	0.25	0	0	0.111	0
**BrcSpond**	0.7575	0.8314	55.96	0	0	0	0.444	0.5
**Unio**	0.7493	0.8224	56.78	0.0625	0	0	0.556	0
**CMartP**	0.7008	0.7692	57.55	0	0.154	0.6	0	0
**PCalTub**	0.6862	0.7531	58.3	0	0	0	0.444	0.25
**CMelesP**	0.6816	0.7481	59.05	0.0625	0.0769	0.6	0	0.25
**Potamide**	0.6456	0.7085	59.76	0.188	0	0	0.222	0
**PSheTrg**	0.6452	0.7081	60.47	0.0625	0	0	0.444	0
**BrcCalc**	0.6392	0.7015	61.17	0	0	0	0.444	0.5
**BrcRv**	0.6322	0.6939	61.86	0	0	0	0.444	0.25
**PStoDsc**	0.5737	0.6296	62.49	0.125	0	0	0	0.75
**Patella**	0.5723	0.6282	63.12	0.25	0	0	0	0.5
**CLutP**	0.5666	0.6218	63.74	0	0.154	0.4	0	0
**CAlcP**	0.5417	0.5945	64.34	0	0.231	0.2	0	0
**PtSpo**	0.5337	0.5857	64.92	0	0	0	0.222	0.5
**PtSchi**	0.5175	0.568	65.49	0.0625	0	0.2	0	0.25

Table S1. cont.								
**Taxon**	**Av. dissim**	**Contrib. %**	**Cumulative %**	**WEM**	**BM**	**BEN**	**DEN**	**MEN**
**BrcGly**	0.493	0.5411	66.03	0	0	0	0.222	0.75
**Acantho**	0.4865	0.5339	66.57	0.125	0	0	0	0.75
**IEquP**	0.4808	0.5277	67.09	0	0.154	0.4	0	0
**TGardon**	0.4572	0.5018	67.6	0.188	0	0	0	0
**CPhoP**	0.4385	0.4813	68.08	0	0.0769	0.4	0	0
**PSheRd**	0.405	0.4445	68.52	0.0625	0	0	0.111	0
**PSheGtt**	0.3969	0.4357	68.96	0	0	0	0.222	0.5
**BrcArg**	0.3908	0.429	69.39	0	0	0	0.333	0
**PSheTrz**	0.3908	0.429	69.81	0	0	0	0.333	0
**BrcOs**	0.3889	0.4268	70.24	0	0.0769	0	0.111	0
**Veneri**	0.3805	0.4177	70.66	0	0	0	0.333	0
**PSchiDsc**	0.3697	0.4058	71.06	0.0625	0	0	0	0.5
**ICaprP**	0.3674	0.4032	71.47	0	0.154	0	0	0.25
**AnShell**	0.3652	0.4009	71.87	0	0	0	0	1
**Ostrea**	0.3599	0.395	72.26	0.188	0	0	0	0
**Conus**	0.3566	0.3914	72.66	0.125	0	0	0	0.75
**EnaDetrita**	0.3475	0.3814	73.04	0	0	0	0.222	0
**BrcMbr**	0.3353	0.368	73.4	0	0	0	0.111	0.5
**Luria**	0.3312	0.3635	73.77	0.0625	0	0	0	0.5
**MCanP**	0.3242	0.3558	74.12	0	0.0769	0.2	0	0.25
**PlOs**	0.3232	0.3548	74.48	0	0.0769	0	0	0.75
**IUrsP**	0.3083	0.3384	74.82	0	0.0769	0.2	0	0
**MCastP**	0.2996	0.3289	75.15	0	0.0769	0.2	0	0
**BrcGres**	0.2939	0.3226	75.47	0	0	0	0.222	0
**Ampulli**	0.2936	0.3223	75.79	0.188	0	0	0	0
**PRVOv**	0.2935	0.3221	76.11	0	0	0	0	0.75
**BrcVC**	0.2926	0.3211	76.43	0	0	0	0.222	0
**CEqP**	0.291	0.3194	76.75	0	0	0.4	0	0
**PMSusP**	0.291	0.3194	77.07	0	0	0.4	0	0
**PMAlcP**	0.291	0.3194	77.39	0	0	0.4	0	0
**PtGly**	0.2858	0.3136	77.71	0.0625	0	0	0	0.5
**MHumP**	0.284	0.3117	78.02	0.0625	0.154	0	0	0
**PCalGtt**	0.2822	0.3097	78.33	0	0	0	0.111	0.5
**Melanopsis**	0.2818	0.3092	78.64	0.0625	0	0	0	0.25
**PArgTub**	0.2817	0.3092	78.95	0	0	0	0	0.75
**PSttDsc**	0.2817	0.3092	79.26	0	0	0	0	0.75
**PStoTub**	0.2808	0.3082	79.56	0.0625	0	0	0.111	0.25
**Turritel**	0.2746	0.3014	79.86	0.0625	0	0	0.111	0
**Mytilus**	0.2676	0.2937	80.16	0.0625	0	0	0.111	0.25
**MPhoP**	0.2624	0.288	80.45	0	0.154	0	0	0
**IHumP**	0.2624	0.288	80.73	0	0.154	0	0	0
**IMelP**	0.2624	0.288	81.02	0	0.154	0	0	0
**Ocenebra**	0.2616	0.2872	81.31	0.188	0	0	0	0
**Laevicar**	0.2616	0.2872	81.6	0.188	0	0	0	0
**PendCastP**	0.2584	0.2836	81.88	0	0.0769	0.2	0	0
**PtQrtz**	0.2576	0.2827	82.16	0.0625	0.0769	0	0	0.25
**MAlcP**	0.2496	0.274	82.44	0	0.0769	0.2	0	0
**PCalCar**	0.2387	0.262	82.7	0	0	0	0.222	0
**Cypraea**	0.2367	0.2598	82.96	0.0625	0	0	0	0.5
**Gyraulus**	0.2352	0.2581	83.22	0.125	0	0	0	0
**IRoeDeerP**	0.2282	0.2505	83.47	0	0.154	0	0	0
**Pirenella**	0.2251	0.2471	83.71	0.125	0	0	0	0
**PLignDsc**	0.222	0.2436	83.96	0	0	0	0.222	0
**Aporrhai**	0.2211	0.2426	84.2	0.0625	0	0	0	0.25
**PSerpDsc**	0.2188	0.2402	84.44	0	0	0	0	0.5
**Charo**	0.2181	0.2394	84.68	0	0	0	0	0.75
**Osilinus**	0.2145	0.2354	84.92	0.0625	0	0	0	0.25
**BrcRn**	0.2123	0.233	85.15	0	0	0	0.111	0.25
**Bayania**	0.2116	0.2323	85.38	0.125	0	0	0	0
**IVulpP**	0.2108	0.2313	85.61	0	0.154	0	0	0

Table S1. cont.								
**Taxon**	**Av. dissim**	**Contrib. %**	**Cumulative %**	**WEM**	**BM**	**BEN**	**DEN**	**MEN**
**BrcOsili**	0.21	0.2305	85.84	0	0	0	0	0.5
**spisula**	0.2096	0.23	86.07	0.0625	0	0	0.111	0
**Phalium**	0.2018	0.2215	86.29	0.0625	0	0	0	0.5
**PlSpoAt**	0.1956	0.2147	86.51	0	0	0	0.111	0
**McelP**	0.1819	0.1996	86.71	0.0625	0.0769	0	0	0
**Euspira**	0.1796	0.1972	86.91	0.125	0	0	0	0
**Chlamys**	0.1765	0.1937	87.1	0.0625	0	0	0	0.25
**Trophon**	0.168	0.1844	87.28	0.0625	0	0	0	0.25
**Haliotis**	0.168	0.1844	87.47	0.0625	0	0	0	0.25
**MLutrGr**	0.1657	0.1819	87.65	0	0.0769	0	0	0
**POsD**	0.1657	0.1819	87.83	0	0.0769	0	0	0
**PMUrsGr**	0.1657	0.1819	88.01	0	0.0769	0	0	0
**PtAnt**	0.1649	0.1809	88.2	0.0625	0.0769	0	0	0
**PMudDsc**	0.1611	0.1768	88.37	0.0625	0	0	0	0
**AnSchi**	0.1575	0.1729	88.54	0	0	0	0.111	0.25
**CFelP**	0.1571	0.1724	88.72	0	0.0769	0	0	0.25
**PtCal**	0.1552	0.1704	88.89	0	0	0	0	0.5
**PhalCelP**	0.1539	0.169	89.06	0.125	0	0	0	0
**Macoma**	0.1532	0.1681	89.22	0.0625	0	0	0	0
**IPhoP**	0.1501	0.1647	89.39	0	0	0.2	0	0
**PArgRd**	0.1471	0.1614	89.55	0	0	0	0	0.25
**Stud**	0.1471	0.1614	89.71	0	0	0	0	0.25
**PtStt**	0.1471	0.1614	89.87	0	0	0	0	0.25
**PtSto**	0.1471	0.1614	90.04	0	0	0	0	0.25
**PtMbr**	0.1471	0.1614	90.2	0	0	0	0	0.25
**PRVTub**	0.1471	0.1614	90.36	0	0	0	0	0.25
**PRVBout**	0.1471	0.1614	90.52	0	0	0	0	0.25
**PMbrDsc**	0.1471	0.1614	90.68	0	0	0	0	0.25
**PGalDsc**	0.1471	0.1614	90.84	0	0	0	0	0.25
**PGalBic**	0.1471	0.1614	91	0	0	0	0	0.25
**Psteatdsc**	0.1464	0.1607	91.16	0	0	0	0	0.5
**AnCelAnt**	0.1464	0.1607	91.33	0	0	0	0	0.5
**POsDsc**	0.1464	0.1607	91.49	0	0	0	0	0.5
**DisStoP**	0.1464	0.1607	91.65	0	0	0	0	0.5
**DisOsP**	0.1464	0.1607	91.81	0	0	0	0	0.5
**Clancu**	0.1461	0.1603	91.97	0.0625	0	0	0	0
**Callista**	0.1461	0.1603	92.13	0.0625	0	0	0	0
**Tympano**	0.1418	0.1556	92.28	0	0	0	0.111	0
**PSheOvBi**	0.1418	0.1556	92.44	0	0	0	0.111	0
**Pisania**	0.1412	0.155	92.59	0.0625	0	0	0	0.25
**AstrFelP**	0.141	0.1547	92.75	0	0	0.2	0	0
**COttP**	0.141	0.1547	92.9	0	0	0.2	0	0
**AstrCasP**	0.141	0.1547	93.06	0	0	0.2	0	0
**EmOrbi**	0.141	0.1547	93.21	0	0	0.2	0	0
**Conorbis**	0.1397	0.1533	93.37	0.0625	0	0	0	0
**Keilosto**	0.1397	0.1533	93.52	0.0625	0	0	0	0
**Amalda**	0.1397	0.1533	93.67	0.0625	0	0	0	0
**PSheRectBip**	0.1372	0.1506	93.82	0	0	0	0.111	0
**Pnéphrite**	0.1372	0.1506	93.97	0	0	0	0.111	0
**Pmbr**	0.1372	0.1506	94.12	0	0	0	0.111	0
**Strombus**	0.1346	0.1478	94.27	0	0	0	0	0.5
**PCalOv**	0.1346	0.1478	94.42	0	0	0	0	0.5
**Stramoni**	0.1346	0.1478	94.57	0	0	0	0	0.5
**Nerita**	0.125	0.1372	94.71	0	0	0	0.111	0
**Corbicul**	0.125	0.1372	94.84	0	0	0	0.111	0
**Stenomph**	0.125	0.1372	94.98	0	0	0	0.111	0
**PtUnio**	0.125	0.1372	95.12	0	0	0	0.111	0
**PSheCrrMl**	0.125	0.1372	95.25	0	0	0	0.111	0
**PSheCrrBi**	0.125	0.1372	95.39	0	0	0	0.111	0
**BrcAntl**	0.125	0.1372	95.53	0	0	0	0.111	0

Table S1. cont.								
**Taxon**	**Av. dissim**	**Contrib. %**	**Cumulative %**	**WEM**	**BM**	**BEN**	**DEN**	**MEN**
**Granulo**	0.125	0.1372	95.67	0	0	0	0.111	0
**MandiCastP**	0.1141	0.1252	95.79	0	0.0769	0	0	0
**MandiAlcPerf**	0.1141	0.1252	95.92	0	0.0769	0	0	0
**ILagomP**	0.1141	0.1252	96.04	0	0.0769	0	0	0
**ICastPl**	0.1141	0.1252	96.17	0	0.0769	0	0	0
**PhalOursgorge**	0.1141	0.1252	96.29	0	0.0769	0	0	0
**PCastP**	0.1141	0.1252	96.42	0	0.0769	0	0	0
**dentRequin**	0.1141	0.1252	96.54	0	0.0769	0	0	0
**Buccinum**	0.1077	0.1182	96.66	0.0625	0	0	0	0
**Nerinea**	0.09693	0.1064	96.77	0	0	0	0.111	0
**POsTrz**	0.09693	0.1064	96.87	0	0	0	0.111	0
**PosBic**	0.09693	0.1064	96.98	0	0	0	0.111	0
**BrcVCTrP**	0.09693	0.1064	97.09	0	0	0	0.111	0
**CPutP**	0.09668	0.1061	97.19	0	0.0769	0	0	0
**Marginel**	0.0835	0.09164	97.28	0	0	0	0	0.25
**PVascOv**	0.0835	0.09164	97.38	0	0	0	0	0.25
**Gibberul**	0.0835	0.09164	97.47	0	0	0	0	0.25
**DisNacrP**	0.0835	0.09164	97.56	0	0	0	0	0.25
**AnCalc**	0.0835	0.09164	97.65	0	0	0	0	0.25
**TmamSeaGr**	0.082	0.09001	97.74	0.0625	0	0	0	0
**PlCalcAt**	0.082	0.09001	97.83	0.0625	0	0	0	0
**Bithynia**	0.082	0.09001	97.92	0.0625	0	0	0	0
**Venus**	0.07194	0.07895	98	0.0625	0	0	0	0
**Fagotia**	0.07194	0.07895	98.08	0.0625	0	0	0	0
**Calliost**	0.07194	0.07895	98.16	0.0625	0	0	0	0
**Melongen**	0.07174	0.07873	98.24	0	0	0	0	0.25
**ArcaNoae**	0.07174	0.07873	98.32	0	0	0	0	0.25
**PSiliDsc**	0.07174	0.07873	98.39	0	0	0	0	0.25
**POsTrg**	0.07174	0.07873	98.47	0	0	0	0	0.25
**POsRect**	0.07174	0.07873	98.55	0	0	0	0	0.25
**POsMola**	0.07174	0.07873	98.63	0	0	0	0	0.25
**POsCra**	0.07174	0.07873	98.71	0	0	0	0	0.25
**Pophio**	0.07174	0.07873	98.79	0	0	0	0	0.25
**BolmOper**	0.07174	0.07873	98.87	0	0	0	0	0.25
**PGrDsc**	0.07174	0.07873	98.94	0	0	0	0	0.25
**BolmaRug**	0.07174	0.07873	99.02	0	0	0	0	0.25
**DisArgP**	0.07174	0.07873	99.1	0	0	0	0	0.25
**MpodCanP**	0.06291	0.06904	99.17	0	0	0	0	0.25
**Scaphand**	0.06291	0.06904	99.24	0	0	0	0	0.25
**PtPect**	0.06291	0.06904	99.31	0	0	0	0	0.25
**CLynxP**	0.06291	0.06904	99.38	0	0	0	0	0.25
**PtGr**	0.06291	0.06904	99.45	0	0	0	0	0.25
**AnMbr**	0.06291	0.06904	99.52	0	0	0	0	0.25
**BrcCharo**	0.06291	0.06904	99.59	0	0	0	0	0.25
**PRVDsc**	0.06291	0.06904	99.65	0	0	0	0	0.25
**POsOv**	0.06291	0.06904	99.72	0	0	0	0	0.25
**POsGtt**	0.06291	0.06904	99.79	0	0	0	0	0.25
**Hyniapfeifferi**	0.06291	0.06904	99.86	0	0	0	0	0.25
**CardBip**	0.06291	0.06904	99.93	0	0	0	0	0.25
**Donacill**	0.06291	0.06904	100	0	0	0	0	0.25

Table B. List of the shell taxa used as beads and recorded in the database

Valid name		Class
**Marin shells**		
Acanthocardia echinata	Linné, 1758	Bivalvia
Acanthocardia	Linné, 1758	Bivalvia
Arca noae	Linné, 1758	Bivalvia
Callista chione	Linné, 1758	Bivalvia
Cardita sp.	Bruguiére, 1792	Bivalvia
Cardium sp.	Linné, 1758	Bivalvia
Cerastoderma edule	Linné, 1758	Bivalvia
Mimachlamys varia	Linné, 1758	Bivalvia
Donacilla cornea	Poli, 1791	Bivalvia
Donax trunculus	Linné, 1758	Bivalvia
Glycymeris glycymerys	Linné, 1758	Bivalvia
Glycymeris sp.	da Costa, 1778	Bivalvia
Glycymeris	Lamarck, 1819	Bivalvia
Laevicardium crassum	Gmelin, 1791	Bivalvia
Macoma balthica	Linné, 1758	Bivalvia
Mytilus edulis	Linné, 1758	Bivalvia
Mytilus sp.	Linné, 1758	Bivalvia
Ostrea edulis	Linné, 1758	Bivalvia
Pecten Maximus	Linné, 1758	Bivalvia
Pecten sp.	Müller, 1776	Bivalvia
Spisula sp.	Linné, 1758	Bivalvia
Spondylus gaederopus	Linné, 1758	Bivalvia
Venericardia antiquata	Linné, 1758	Bivalvia
Venus verrucosa	Linné, 1758	Bivalvia
Aporrhais pespelecani	Linné, 1758	Gastropoda
Architectonica sp.	Röding, 1798	Gastropoda
Bolma rugosa	Linné, 1767	Gastropoda
Buccinum undatum	Linné, 1758	Gastropoda
Calliostoma zizyphinum	Linné, 1758	Gastropoda
Cerithium sp.	Bruguière, 1789	Gastropoda
Cerithium vulgatum	Bruguière, 1792	Gastropoda
Charonia lampas	Linné, 1758	Gastropoda
Clanculus corallinus	Gmelin, 1791	Gastropoda
Columbella rustica	Linné, 1758	Gastropoda
Conus mediterraneus	Hwass in Bruguière,	Gastropoda
Conus sp.	Linné, 1758	Gastropoda
Cyclope neritea	Linné, 1758	Gastropoda
Cyclope sp.	Linné, 1758	Gastropoda
Euspira catena	da Costa 1778	Gastropoda
Euspira pulchella	Risso, 1826	Gastropoda
Euspira sp.	Risso, 1826	Gastropoda
Gibberula miliaria	Linné, 1758	Gastropoda
Haliotis sp.	Linné, 1758	Gastropoda
Homalopoma	Linné, 1758	Gastropoda
Littorina fabalis	Turton, 1825	Gastropoda
Littorina littorea	Linné, 1758	Gastropoda
Littorina obtusata	Linné, 1758	Gastropoda
Littorina sp.	Ferussac, 1822	Gastropoda
Luria lurida	Linné, 1758	Gastropoda
Marginella sp.	Lam, 1799	Gastropoda
Mitra sp.	Linné, 1758	Gastropoda
Nassarius corniculum	Olivi, 1792	Gastropoda
Nassarius cuvierii	Payraudeau, 1826	Gastropoda
Nassarius gibbosulus	Linné, 1758	Gastropoda
Nassarius incrassatus	Ström, 1768	Gastropoda
Nassarius mutabilis	Linné, 1758	Gastropoda
Nassarius nitidus	Jeffreys, 1867	Gastropoda
Nassarius reticulatus	Linné, 1758	Gastropoda
Nassarius pfeifferi	Philippi, 1844	Gastropoda
Natica sp.	Scopoli, 1777	Gastropoda
Natica	Gemlin, 1791	Gastropoda
Nerita tricarinata	Linné, 1758	Gastropoda
Nucella lapillus	Linné, 1758	Gastropoda
Ocenebra erinaceus	Linné, 1758	Gastropoda
Osilinus sp.	Lamarck, 1822	Gastropoda
Osilinus turbinatus	Born, 1778	Gastropoda
Patella caerulea	Linné, 1758	Gastropoda
Patella sp.	Linné, 1758	Gastropoda
Patella vulgata	Linné, 1758	Gastropoda
Phalium saburon	Bruguière, 1792	Gastropoda
Pisania striata	Gmelin, 1791	Gastropoda
Ruditapes sp.	Linné, 1758	Gastropoda
Scaphander lignarius	Linné, 1758	Gastropoda
Stramonita haemastom	Linné, 1767	Gastropoda
Strombus sp.	Linné, 1758	Gastropoda
Trivia monacha	da Costa 1778	Gastropoda
Trivia sp.	da Costa 1778	Gastropoda
Trophonopsis muricatus	Montagu, 1803	Gastropoda
Turritella communis	Risso, 1826	Gastropoda
Turritella sp.	Lamarck, 1799	Gastropoda

Valid name		Class
**Freshwater shells**		
Anodonta sp.	Lamarck, 1799	Bivalvia
Margaritifera sp.	Schumacher, 1816	Bivalvia
Unio sp.	Philipsson, 1788	Bivalvia
Bithynia tentaculata	Linnaeus, 1758	Gastropoda
Esperiana sp.	Férussac , 1823	Gastropoda
Lithoglyphus naticoides	Pfeiffer, 1828	Gastropoda
Melanopsis sp.	Férussac, 1807	Gastropoda
Theodoxus danubialis	Pfeiffer, 1828	Gastropoda
Theodoxus fluviatilis	Linné, 1758	Gastropoda
**Terrestrial shells**		
Zebrina detrita	Müller, 1774	Gastropoda
Discus rotundatus	Müller, 1774	Gastropoda
**Fossil shells**		
Corbicula convexa	Deshayes, 1854	Bivalvia
Spisula solida	Linné, 1758	Bivalvia
Glycymeris obovatus	Lamarck, 1805	Bivalvia
Glycymeris pulvinata	Lamarck, 1805	Bivalvia
Pinctada sp.	Röding, 1798	Bivalvia
Isognomon maxillata	Lamarck, 1819	Bivalvia
Ampullina sp.	Férussac 1822	Gastropoda
Bayania lactea	Lamarck, 1806	Gastropoda
Granulolabium Plicatum	Bruguiére, 1792	Gastropoda
Conorbis sp.	Swainson, 1840	Gastropoda
Gyraulus trochiformis	Rafinesque, 1815	Gastropoda
Gyraulus sulcatus	Hilgendorf, 1866	Gastropoda
Keilostoma sp.	Deshayes, 1850	Gastropoda
Nerinea sp.	Defrance, 1825	Gastropoda
Amalda sp.	Adams et Adams,	Gastropoda
Potamides laevissimus	Sanberger, 1863	Gastropoda
Potamides lamarckii	Brongniart, 1810	Gastropoda
Potamides plicatus	Brongniart, 1810	Gastropoda
Potamides sp.	Brongniart, 1810	Gastropoda
Trophon fusulus	Brocchi, 1814	Gastropoda
Stenomphalus	Sandberger, 1853	Gastropoda
Theodoxus greagrius	Thomä, 1845	Gastropoda
Tympanotonos sp.	Linné, 1758	Gastropoda
Dentalium sp.	Linné, 1758	Scaphopoda
Distrupa sp.	Berkeley, 1835	Canalipalpat
Protula sp.	Risso, 1826	Polychaeta

Table C. List of the vertebrate bones and teeth recorded in the database (I: incisor, C: canine, M: molar, indet: indeterminate)

Family		Skeletal part
Bovidae		
	Bos sp.	I
	Capra ibex	I
	Caprinae indet.	I
Cervidae		
	Alces alces	I, C, PM, M, mandible
	Capreolus capreolus	I, C
	Cervus elaphus	I, C, M, antler, phalanx
Suidae		
	Sus scrofa	I, C, PM
Equidae		
	Equus ferus	I, C
Castoridae		
	Castor fiber	I, M, astragalus, mandible
Mustelidae		
	Lutra lutra	C, M
	Martes martes	C
	Meles meles	I, C
	Mustela putorius	C
Felidae		
	Felis silvestris	C, astragalus
	Lynx sp.	C
Canidae		
	Vulpes vulpes	I, C
	Canis familiaris	I, C, M
	Canis lupus	I, C, M
Ursidae		
	Ursus arctos	I, C
Phocidae		
	indet	I, C
Cyprinidae		
	Rutilus sp.	Pharyngeal teeth
Hominidae		
	Homo sapiens sapiens	I, C, M
Leporidae		
	indet	long bone
**Bird**	indet	long bone
**Fish**	indet	vertebra
	Rutilus sp.	pharyngeal tooth
	Shark	tooth
**Tortoise**	*Emys orbicularis*	scale

Table D. List of the mineral raw materials recorded in the database

Type of mineral raw material	Sub-types
Clay	
Limestone	
Marble	
Soapstone	
Schist	
Sandstone	
Siltstone	
Quartz	
**Grey stone**	
**Green stone**	Serpentine
	Jade
	Amphibolite
	Hornblendite
	Pyroxenite
	Phyllite
	Nephrite
	Variscite
**Black stone**	Lignite
	Ophiolite
**Granitic rock**	
**Pebble**	Indet

Table E. List of the bead-types recorded in the database

Bead-type	Type description
Acantho	Acanthocardia sp.
Ampulli	Ampullina sp.
AnCalc	Limestone ring
AnCelAnt	Red deer antler ring
AnMbr	Marble ring
AnOs	Bone ring
AnSchi	Schist ring
AnShell	Shell ring
Antalis	Antalis sp.
Aporrhai	Aporrhais pespelecani
ArcaNoae	Arca noae
AstrCasP	Beaver Astragalus
AstrFelP	Felis astragalus
Bayania	Bayania sp.
Bithynia	Bithynia tentaculata
BolmaRug	Bolma rugosa
BolmOper	Bolma rugosa opercule
BrcAntl	Red deer antler bracelet
BrcArg	Clay bracelet
BrcCalc	Limestone bracelet
BrcCharo	Charonia lampas bracelet
BrcGly	Glycymeris bracelet
BrcGres	Sandstone bracelet
BrcMbr	Marble bracelet
BrcOs	Bone bracelet
BrcOsili	Osilinus bracelet
BrcRn	Black stone bracelet
BrcRv	Green stone bracelet
BrcSchi	Schist bracelet
BrcSpond	Spondylus bracelet
BrcVC	Green stone bracelet
BrcVCTrP	Green stone bracelet

Table S5. cont.	
**Bead-type**	**Type description**
Buccinum	Buccinum undatum
CAlcP	Elk canine
Calliost	Calliostoma zizyphinum
Callista	Callista chione
CCanP	Canidae canine
CCelP	Red deer canine
CEqP	Horse canine
Cerast	Cerastoderma sp.
Cerith	Cerithium sp.
CFelP	Felis canine
Charo	Charonia lampas
Chlamys	Chlamys varia
Clancu	Clanculus corallinus
CLutP	Otter canine
CLynxP	Lynx Canine
CMartP	Marten canine
CMelesP	Badger canine
Columbel	Columbella rustica
Conus	Conus sp.
Corbicul	Corbicula convexa
COttP	Otter canine
CPhoP	Seal canine
CPutP	Polecat canine
CSusP	Wild boar canine
CUrsP	Bear canine
CVulP	Fox canine
Cyclope	Cyclope neritea
Cypraea	Cypraea sp.
dentRequin	Shark tooth
DisArgP	Discoïd clay bead
DisNacrP	Discoïd mother of pearl bead
DisOsP	Discoïd bone bead
DisStoP	Discoïd stone bead
Donacill	Donacilla cornea
EmOrbi	Emys orbicularis scale
EnaDetrita	Ena detrita
Euspira	Euspira catena
Fagotia	Fagotia
Gibberul	Gibberula miliaria
Gly	Glycymeris sp.
Granulo	Granulolabium Plicatum
Gyraulus	Gyraulus sp.
Haliotis	Haliotis sp.
Hyniapfeifferi	Hynia pfeifferi
IAlcP	Elk incisor
IBosP	Bovidae incisor
ICanP	Canidae incisor
ICaprP	Ibex incisor
ICastP	Beaver incisor
ICastPl	Beaver incisor
ICelP	Red deer incisor
IEquP	Horse incisor
IHumP	Human incisor
ILagomP	Lagomorph bone
IMelP	Badger incisor
IPhoP	Seal incisor
IRoeDeerP	Roe deer incisor
ISusP	Wild boar incisor
IUrsP	Bear incisor
IVulpP	Fox incisor
Laevicar	Laevicardium sp.
Lithogly	Lithoglyphus naticoides
Littor	Littorina sp.
Luria	Luria lurida
Macoma	Macoma balthica
MAlcP	Elk molar
MandiAlcPerf	Elk mandible
MandiCastP	Beaver mandible
Marginel	Marginella sp.

Table S5. cont.	
**Bead-type**	**Type description**
MCanP	Canidae molar
MCastP	Beaver molar
McelP	Red deer molar
Melanopsis	Melanopsis sp.
Melongen	Melongena sp.
MHumP	Human molar
MLutrGr	Otter molar
MPhoP	Seal molar
MpodCanP	Perforated Canidae metapod
Mytilus	Mytilus sp.
nassariu	Nassurius sp.
Natica	Natica sp.
Nerinea	Nerinea
Nerita	Nerita tricarinata
Nucella	Nucella lapillus
Ocenebra	Ocenebra erinaceus
Osilinus	Osilinus sp.
Ostrea	Ostrea sp.
PAmb	Amber bead
PArgRd	Round clay bead
PArgTub	Tubular clay bead
Patella	Patella sp.
PCalCar	Square limestone bead
PCalDsc	Discoïd limestone bead
PCalGtt	Limestone bead with circular section and curved profile
PCalOv	Oval limestone bead
PCalTub	Tubular limestone bead
PCastP	Beaver incisor
Pecten	Pecten sp.
PendCastP	Beaver bone
PGalBic	Pebble pendant
PGalDsc	Discoïd stone bead
PGrDsc	Discoïd sandstone bead
PhalCelP	Red deer phalanx
Phalium	Phalium saburon
PhalOursgorge	Bear phalanx
Pirenella	Pirenella sp.
Pisania	Pisania striata
PlCalcAt	Limestone plate
PLignDsc	Discoidal black stone bead
PlOs	Bone plate
PlSpoAt	Spondylus plate
PMAlcP	Elk premolar
Pmbr	Round amber bead
PMbrDsc	Discoïd marble bead
PMSusP	Wild boar premolar
PMudDsc	Discoidal clay bead
PMUrsGr	Grooved bear molar
Pnéphrite	Discoid black stone bead
Pophio	Discoid black stone bead
PosBic	Biconic bone bead
POsCra	Bone bead red deer canine shaped
POsD	Bone pendant
POsDsc	Discoïd bone bead
POsGtt	Bone bead with circular section and curved profile
POsMola	Bone bead mammal molar shaped
POsOv	Oval bone bead
POsRect	Rectangular bone bead
POsTrg	Trivangular Bone Bead
POsTrz	Trapezoidal shell bead
POsTub	Tubular bone bead
Potamide	Potamides sp.
PRVBout	Round green stone bead
PRVDsc	Discoïd green stone bead
PRVOv	Oval green stone bead
PRVTub	Tubular green stone bead
PSchiDsc	Discoïd schist bead
PSerpDsc	Discoid green stone bead
PSheCrrBi	Square shell bead

Table S5. cont.	
**Bead-type**	**Type description**
PSheCrrMl	Square shell bead
PSheDsc	Discoïd shell bead
PSheGtt	Shell bead with circular section and curved profile
PSheOv	Oval shell bead
PSheOvBi	Bi-perforated oval shell bead
PSheRd	Round shell bead
PSheRectBip	Restangular shell bead
PSheTrg	Triangular shell bead
PSheTrz	Trapezoidal shell bead
PSiliDsc	Discoïd siltstone bead
PSpoTub	Tubular spondylus bead
Psteatdsc	Discoïd soapstone bead
PStoDsc	Discoïd stone bead
PStoTub	Tubular limestone bead
PSttDsc	Discoïd soapstone bead
PtAmb	Amber pendant
PtAnt	Red deer antler pendant
PtCal	Limestone pendant
PtGal	Pebble pendant
PtGly	Glycymaris pendant
PtGr	Sandstone pendant
PtMbr	Marble pendant
PtOs	Bone pendant
PtPect	Pecten pendant
PtQrtz	Quartz pendant
PtSchi	Schist pendant
PtSpo	Arciform Spondylus pendant
PtSto	Stone pendant
PtStt	Soapstone pendant
PtUnio	Unio sp.
PVascOv	Oval green stone bead
Scaphand	Scaphander lignarius
spisula	Spisula solida
SpoInc	Notched Spondylus
Spond	Spondylus sp.
Stenomph	Stenomphalus
Stramoni	Stramonita haemastoma
Strombus	Strombus sp.
Stud	Clay ear stud
TGardon	Rutilus pharyngeal tooth
Theodoxu	Theodoxus sp.
TmamSeaGr	Grooved Seal canine
Trivia	Trivia sp.
Trophon	Trophonopsis muricatus
Turritel	Turritella sp.
Tympano	Tympanotonos sp.
Unio	Unio sp.
Veneri	Venericardia antiquata
Venus	Venus verrucosa

Table F. List of the bead-types present in each of the archaeological cultures

Archaeological culture	Bead-types
1	AnOs, Antalis, BrcArg, BrcCalc, BrcGres, BrcSchi, Cerast, CSusP, Mytilus, PCalDsc, PCalGtt, PSheDsc, PSheTrz, PSpoTub, spisula, SpoInc, Spond, Turritel, Tympano, Unio, Veneri
2	AnOs, Antalis, BrcArg, BrcCalc, BrcGres, BrcRn, BrcRv, BrcSchi, CSusP, Natica, Nucella, PCalDsc, PCalTub, POsTub, PSheDsc, PSheTrz, PSpoTub, PtGal, Unio
3	AnOs, Antalis, BrcGly, PCalDsc, PCalTub, PSheDsc, PSheOv, PSheRd, PSpoTub, PtOs, SpoInc, Spond, Theodoxu
4	AnOs, Antalis, BrcRv, BrcSchi, EnaDetrita, Nucella, PCalDsc, Pecten, POsTub, PSheDsc, PSheOv, PSheTrg
5	AnOs, AnSchi, Antalis, BrcArg, BrcCalc, BrcGly, BrcRv, BrcSchi, BrcSpond, BrcVC, BrcVCTrP, CCanP, CCelP, Cerith, CSusP, Cyclope, Littor, Natica, Nerinea, Nucella, PCalCar, PCalDsc, PLignDsc, PosBic, POsTrz, PSheDsc, PSheOv, PSheTrg, PSheTrz, PSpoTub, PtGal, SpoInc, Spond, Trivia, Veneri
6	Antalis, BrcAntl, BrcCalc, BrcRv, BrcSchi, CCanP, CCelP, Corbicul, CSusP, Gly, Granulo, Nerita, PCalDsc, PLignDsc, Potamide, PSheCrrBi, PSheCrrMl, PSheDsc, PSheGtt, PSheOv, PtUnio, Spond, Stenomph, Theodoxu, Unio
7	Antalis, BrcSpond, CCelP, Columbel, CSusP, CVulP, Lithogly, PCalTub, Pmbr, Pnéphrite, PSheDsc, PSheGtt, PSheOv, PSheRectBip, PSheTrg, PSpoTub, PStoTub, PtSpo, SpoInc, Spond, Theodoxu, Unio
8	Antalis, BrcMbr, BrcOs, BrcSpond, BrcVC, Gly, PlSpoAt, Potamide, PSheOv, PSpoTub, PtSpo, SpoInc, Spond
9	AnOs, Antalis, BrcSpond, CCelP, Columbel, EnaDetrita, Gly, Natica, Nucella, PCalCar, PCalTub, PSheDsc, PSheOv, PSheOvBi, PSheTrg, PSpoTub, SpoInc, Spond, Trivia, Unio, Veneri
10	AnShell, Antalis, BrcOsili, BrcSpond, PArgRd, PArgTub, PGalBic, PGalDsc, PMbrDsc, POsTub, PRVBout, PRVOv, PRVTub, PSchiDsc, PSerpDsc, PSheDsc, PStoDsc, PSttDsc, PtMbr, PtSpo, PtSto, PtStt, Spond, Stud
11	Acantho, AnShell, Antalis, ArcaNoae, BolmaRug, BolmOper, BrcGly, BrcSpond, CCanP, CCelP, Cerast, Cerith, Charo, Chlamys, Columbel, Conus, CSusP, CUrsP, CVulP, Cyclope, Cypraea, DisArgP, Gly, IBosP, ICaprP, MCanP, Melongen, Osilinus, PArgTub, Patella, PCalDsc, PCalOv, PCalTub, Pecten, PGrDsc, Phalium, Pisania, PlOs, Pophio, POsCra, POsMola, POsRect, POsTrg, POsTub, PSchiDsc, PSerpDsc, PSheDsc, PSheOv, PSiliDsc, PStoTub, PSttDsc, PtCal, PtOs, PtQrtz, PtSpo, SpoInc, Spond, Stramoni, Strombus, Trivia
12	Acantho, AnCelAnt, AnMbr, AnOs, AnSchi, AnShell, Antalis, Aporrhai, BrcCalc, BrcCharo, BrcGly, BrcMbr, BrcOsili, BrcRn, BrcRv, BrcSchi, CardBip, CCanP, CCelP, Cerast, Cerith, CFelP, Charo, CLynxP, Columbel, Conus, CSusP, CVulP, Cyclope, Cypraea, DisOsP, DisStoP, Donacill, Gly, Haliotis, Hyniapfeifferi, Luria, Melanopsis, MpodCanP, Mytilus, nassariu, PArgTub, PCalDsc, PCalGtt, PCalOv, Pecten, Phalium, PlOs, POsDsc, POsGtt, POsOv, POsTub, PRVDsc, PRVOv, PSheDsc, PSheGtt, PSheOv, Psteatdsc, PStoDsc, PSttDsc, PtGly, PtGr, PtOs, PtPect, Scaphand, Stramoni, Strombus, Theodoxu, Trivia, Trophon
13	Acantho, AnCalc, AnCelAnt, AnOs, AnShell, Antalis, BrcCalc, BrcGly, BrcMbr, BrcSchi, CCelP, Cerast, Cerith, Charo, CMelesP, Columbel, Conus, CSusP, CVulP, DisNacrP, DisOsP, DisStoP, Gibberul, Gly, ICelP, ISusP, Luria, Marginel, nassariu, Patella, PCalDsc, PCalGtt, Pecten, PlOs, POsDsc, POsTub, PRVOv, PSheDsc, PSheGtt, PSheOv, Psteatdsc, PStoDsc, PtCal, PtGal, PtGly, PtOs, PtSchi, PVascOv, Theodoxu,, Trivia
14	BrcGly, BrsSpond
15	Cyclope, Lithogly, Melanopsis, PCalDsc, PSheRd, TGardon
16	Acantho, CSusP, Littor, Nucella, Ostrea, Patella, Pecten, PtGal, Trivia
17	Ampulli, Antalis, Bayania, Calliost, CCanP, CCelP, Cerast, Cerith, CMelesP, Columbel, Conus, CVulP, Euspira, Fagotia, Gly, ICelP, Laevicar, McelP, Mytilus, nassariu, Natica, Ocenebra, Ostrea, Patella, Pecten, PhalCelP, Phalium, Pirenella, Pisania, Potamide, PtAnt, PtGal, PtOs, spisula, Theodoxu, Unio, Venus
18	CCelP, Cerast, Columbel, CVulP, Gyraulus, Lithogly, Macoma, Pirenella, POsTub, Potamide, PtOs, TGardon
19	Littor, nassariu, Natica, PtOs, Trivia
20	Aporrhai, CCelP, ICelP, Littor, nassariu, PMudDsc, POsTub, PSchiDsc, PStoDsc, PtAmb, Trivia
21	CCelP, Cerith, Columbel, Cyclope, Lithogly, Luria, nassariu, Theodoxu
22	Antalis, Callista, CCelP, Cerast, Cerith, Columbel, Littor, nassariu, Natica, Osilinus, Pecten, Theodoxu, Trivia
23	Antalis, CCelP, Cerith, Clancu, Columbel, Cyclope, Gly, nassariu, PStoDsc, PtGly, Spond, Theodoxu, Trivia
24	Columbel, nassariu, Natica, Pecten
25	Antalis, Cerith, Columbel, Gly, Pecten
26	Antalis, Columbel, Cyclope, Gly
27	Antalis, Columbel, Cyclope, Gly, PtGal
28	Amalda, Ampulli, Antalis, Bayania, CCelP, Cerast, Conorbis, Gly, Keilosto, Littor, nassariu, Nucella, Potamide
29	Ampulli, Antalis, Bithynia, CCelP, Cerast, Cerith, Columbel, Conus, CVulP, Cyclope, Gyraulus, Laevicar, Lithogly, Littor, MHumP, nassariu, Ocenebra, Patella, Pecten, PhalCelP, PlCalcAt, PSheTrg, PStoTub, PtGal, PtOs, PtQrtz, PtSchi, TGardon, Theodoxu, TmamSeaGr, Trivia
30	Acantho, Antalis, Buccinum, CCelP, Cerast, Chlamys, Cyclope, Cypraea, Euspira, Gly, Haliotis, Laevicar, Littor, nassariu, Nucella, Ocenebra, Ostrea, Patella, Pecten, Trivia, Trophon
31	CCanP, CEqP, CLutP, CMartP, CMelesP, CPhoP, CSusP, CUrsP, CVulP, IAlcP, IBosP, ICanP, ICelP, IEquP, IPhoP, ISusP, IUrsP, MCanP, PAmb, PendCastP, PMAlcP, PMSusP, PtAmb
32	AstrCasP, AstrFelP, CCanP, CEqP, CLutP, CMartP, CMelesP, COttP, CSusP, CVulP, EmOrbi, IAlcP, IBosP, ICanP, ICastP, ICelP, IEquP, ISusP, MAlcP, MCastP, PMAlcP, PMSusP, PtOs
33	CCanP, CMartP, IAlcP, ICanP, ICastP, IMelP, IUrsP, MCastP, MLutrGr, PMUrsGr, POsD
34	CAlcP, CCanP, CMartP, CMelesP, CPhoP, CUrsP, CVulP, IAlcP, IBosP, ICastP, ICelP, ISusP, PAmb, POsTub, PtAmb
35	CSusP, IAlcP, IBosP, ICelP, ISusP
36	CAlcP, CCelP, CSusP, CUrsP, IAlcP, IBosP, ICelP, IHumP, ISusP, MPhoP, Theodoxu

Table S6. cont.	
Archaeological culture	Bead-types
37	IAlcP, ICelP, PtOs
38	CCelP, CLutP, IAlcP, ICanP, ICelP, Pamb, PtOs
39	BrcOs, CCelP, ICaprP, ICelP, ISusP, PAmb, PtAmb
40	CCelP, ICelP, ISusP
41	CCelP, ICelP, PtAmb
42	CCelP, IAlcP, IBosP
43	ISusP
44	BrcSchi, PAmb, PtAmb, PtSchi
45	CCanP, CUrsP, IAlcP, IBosP, ICanP, ICastP, ICastPl, ICelP, ILagomP, IRoeDeerP, ISusP, IVulpP, MandiAlcPerf, MandiCastP, MCanP, PhalOursgorge, PlOs, POsTub, PtGal, PtOs, PtQrtz
46	CAlcP, CCanP, CCelP, CPhoP, CSusP, CUrsP, dentRequin, IAlcP, IBosP, ICelP, IEquP, IRoeDeerP, ISusP, MAlcP, McelP, MHumP, PAmb, PCastP, PendCastP, POsTub, Theodoxu
47	CAlcP, CCanP, CCelP, CFelP, CLutP, CMartP, CMelesP, CPutP, CSusP, CUrsP, CVulP, IAlcP, IBosP, ICanP, ICaprP, ICastP, ICelP, IEquP, IHumP, IMelP, ISusP, IVulpP, MHumP, MPhoP, PAmb, PtAnt, PtOs
48	IAlcP, ICastP

SUPPORTING DATASET

DATASET S1 (Separate Excel sheet). Database of the Mesolithic and Early Neolithic European sites used in the analysis.

REFERENCES FOR SUPPORTING INFORMATION

1. Dubouloz J. Datation absolue du premier Néolithique du Bassin parisien : complément et relecture des données RRBP et VSG. Bull Soc Prehist fr. 2003; 100: 671–689.

2. Constantin C, Ilett M. Le Néolithique de Villeneuve- St-Germain. Vallée de l'Aisne : cinq années de fouilles protohistoriques. Rev Archéologique Picardie Numéro spécial Numéro spécial. 1982; 1: 121–127.

3. Dubouloz J. Impacts of the Neolithic Demographic Transition on Linear Pottery Culture Settlement. In: Bocquet-Appel J-P, Bar-Yosef O, editors. The Neolithic Demographic Transition and its Consequences. Netherlands: London: Springer Ferlag; 2008. pp. 207–235. Available: http://dx.doi.org/10.1007/978-1-4020-8539-0_9.

4. Allard P. The Mesolithic-Neolithic transition in the Paris Basin: a review. Going Over: the Mesolithic-Neolithic Transition in North-West Europe. Oxford: Alasdair Whittle and Vicki Cummings; 2007. pp. 209–222.

5. Denaire A. Radiocarbon Dating of the Western European Neolithic: Comparison of the Dates on Bones and Dates on Charcoals. Radiocarb. 2001; 51: 657-674. Available: https://journals.uair.arizona.edu/index.php/radiocarbon/article/view/3524.

6. Reingruber A. Early Neolithic settlement patterns and exchange networks in the Aegean. Doc Prehist. 2011; 38: 291–305.

7. Manen C, Philippe S. Chronique radiocarbone de la néolithisation en Méditerranée nord-occidentale. Bull Société Préhistorique Fr. 2003; 100: 479–504.

8. Willigen S van, Hajdas I, Bonani G. New Radiocarbon Dates for the Early Neolithic of the Western Mediterranean. Radiocarb. 2011; 51: 831-838. Available: https://journals.uair.arizona.edu/index.php/radiocarbon/article/view/3537.

9. Biagi P, Spataro M. New observation on the radiocarbon chronology of the starcevo-cris and koros cultures. Prehist Archaeol E Anthropol Theory Educ Rep Prehist Res Proj. 2005; 06-lug: 35–40.

10. Bonsall C, Cook GT, Hedges R, Higham THF, Pickard C, Radovanovic I. Radiocarbon and stable isotope evidence of dietary change from the Mesolithic to the Middle Ages in the Iron Gates : new results from Lepenski Vir. Radiocarbon. 2004; 46: 293–300.

11. Bonsall C. The Mesolithic of the Iron Gates. In: Bailey G, Spikins P, editors. Mesolithic Europe. Cambridge: Cambridge University Press; 2008. pp. 238–279.

12. Bonsall C. "The 'Obanian problem': coastal adaptation in the Mesolithic of western Scotland." In: Pollard T, Morrison A, editors. The Early Prehistory of Scotland. Edinburgh: Edinburgh University Press; 1996. pp. 183–197.

13. Valdeyron N. Le Sauveterrien : cultures et sociétés méso-lithiques dans la France du sud durant le X e et le IX e millénaire BP [mémoire de doctorat nouveau régime, UTM]. Toulouse: Toulouse le Mirail, 1994.

14. Kind C-J. Transport of lithic raw material in the Mesolithic of southwest Germany. J Anthropol Archaeol. 2006; 25: 213–225.

15. Gutiérrez-Zugasti I, Andersen SH, Araújo AC, Dupont C, Milner N, Monge-Soares AM. Shell midden research in Atlantic Europe: State of the art, research problems and perspectives for the future. Shell Midden Res Interdiscip Agenda Quat Soc Sci. 2011; 239: 70–85. doi:10.1016/j.quaint.2011.02.031.

16. Schulting RJ, Richards MP. Dogs, Ducks, Deer and Diet: New Stable Isotope Evidence on Early Mesolithic Dogs from the Vale of Pickering, North-east England. J Archaeol Sci. 2002; 29: 327–333. doi:10.1006/jasc.2002.0707.

17. Olivier Marti B, Tortosa JE, Caballines JJ, Puchol OG, Lopez de Pablo JF. El Mesolítico geométrico de tipo "cocina" en el País Valenciano. In: Utrilla Miranda M del P, Montes L, editors. El mesolítico geométrico en la Península Ibérica. Zaragosa: Universidad de Zaragoza; 2009. pp. 205–258.

18. Tortosa JE, Carrion Marco Y, Puchol OG, Jardon Giner P, Jorda Pardo JF, Balaguer ML, et al. Epipaleolitico-Mesolitico en las Comarcas Centrales Valencianas. In: Alday Ruiz A, editor. El Mesolitico des muescas y denticulados en la cuenca del Ebro y el litoral mediterraneo. Vitoria-Gasteiz: Diputación Foral de Álava; 2006. pp. 65–118.

19. Alday Ruiz A. El Mesolítico de muescas y denticulados en la Cuenca del Ebro y el litoral Mediterráneo peninsular: síntesis de los datos. In: Alday Ruiz A, editor. El mesolítico de muescas y denticulados en la cuenca del Ebro y el litoral mediterráneo peninsular. Vitoria-Gasteiz: Diputación Foral de Álava; 2006. pp. 303–317.

20. Vaquero Rodríguez M. El Mesolítico de Facies Macrolítica en el centro y sur de Cataluña. In: Alday Ruiz A, editor. El mesolítico de muescas y denticulados en la cuenca del Ebro y el litoral mediterráneo peninsular. Vitoria-Gasteiz: Diputación Foral de Álava; 2006. pp. 137–160.

21. Perlès C, Vanhaeren M. Black Cyclope neritea Marine Shell Ornaments in the Upper Palaeolithic and Mesolithic of Franchthi Cave, Greece: Arguments for Intentional Heat Treatment. J Field Archaeol. 2010; 35: 298–309. doi:10.1179/009346910X12707321358874.

22. Ketterer I, Ducrocq T. Le gisement mésolithique du "Petit Marais", La Chaussée-Tirancourt (Somme). Bull Société Préhistorique Fr. 1995; 92: 249–260.

23. Perrin T, Marchand G, Allard P, Binder D, Collina C, Puchol OG, et al. Le second Mésolithique d'Europe occidentale : origines et gradient chronologique. Ann Fond Fyssen. 2009; 24: 160–176.

24. Marchand G. La néolithisation de l'ouest de la France : aires culturelles et transferts techniques dans l'industrie lithique. Bull Société Préhistorique Fr. 2000; 97: 377–403.

25. Eriksson G, Lougas L, Zagorska I. Stone Age hunter–fisher–gatherers at Zvejnieki, northern Latvia: radiocarbon, stable isotope and archaeozoology data. Farming. 2003; 1: 1–25.

26. Stančikaitė M, Baltrūnas V, Šinkūnas P, Kisielienė D, Ostrauskas T. Human response to the Holocene environmental changes in the Biržulis Lake region, NW Lithuania. Quat Int. 2006; 150: 113–129. doi:10.1016/j.quaint.2006.01.010.

27. Casella Colin E, Fowler C. The Archaeology of Plural and Changing Identities : Beyond Identification. New York: Springer; 2005.

28. Terberger T. The Mesolithic Hunter-Fisher-Gatherers on the Northern German Plain. In: Hansen KM, Pedersen KB, editors. Across the western Baltic. Vordingborg: Sydsjællands Museum; 2006. pp. 11–85.

29. Hinz M, Feeser I, Sjögren K-G, Müller J. Demography and the intensity of cultural activities: an evaluation of Funnel Beaker Societies (4200–2800 cal BC). J Archaeol Sci. 2012; 39: 3331–3340. doi:10.1016/j.jas.2012.05.028.

30. Zvelebil M, Dennell RW, Domanska L. Harvesting the sea, farming the forest: the emergence of Neolithic societies in the Baltic region. Sheffiled: Sheffield Academic Press; 1998.

31. Zvelebil M. Innovating hunter-gatherers: the Mesolithic in the Baltic. In: Bailey G, Spikins P, editors. Mesolithic Europe. Cambridge: Cambridge University Press; 2009. pp. 18–59.

32. Velichko AA, Kurenkova EI, Dolukhanov PM. Human socio-economic adaptation to environment in Late Palaeolithic, Mesolithic and Neolithic Eastern Europe. Quat Int. 2009; 1-2: 1-9.

33. Brinch Petersen E, Kozlowski SK. A survey of the Late Palaeolithic and the Mesolithic of Denmark. In: Kozlowski S, editor. The Mesolithic in Europe. Warsaw: Warsaw University Press; 1973. pp. 77–127.

34. Auxiette G. Les bracelets néolithiques dans le Nord de la France, la Belgique et l'Allemagne rhénane. Rev Archéologique Picardie. 1989; 1-2: 13–65.

35. Barge H. Les parures du Néolithique ancien au début de l'Âge des Métaux en Languedoc. Paris: CTHS; 1983.

36. Barge H, Bellier C, Bott S, Cattelain P. Fiches typologiques de l'industrie osseuse préhistorique. Cahier IV : Objets de parure. Aix en Provence: Publication de l'Université de Provence; 1991.

37. Beck CH. Classification and Nomenclature of Beads and Pendants. London: Soc Antiqu London; 1926.

38. Bonnardin S. La parure funéraire au Néolithique ancien dans les Bassins parisiens et rhénans - Rubané, Hinkelstein et Villeneuve-Saint-Germain. 49 M, editor Paris: Mémoire de la Société Préhistorique Française; 2009.

39. Courtin J, Gutherz X. Les bracelets en pierre du Néolithique méridional. Bull Soc Prehist Fr. 1976; 73: 352–369.

40. Praud I. La parure. In: Bostyn F, editor. Néolithique ancien en Haute-Normandie : Le village Villeneuve-Saint-Germain de Poses "Sur la mare" et les sites de la boucle du Vaudreuil. Paris: Société préhistorique française; 2003. pp. 137–256.

41. Taborin Y. La parure en coquilage de l'Epipaléolithique au Bronze ancien en France. Gall Préhistoire. 1974; 17: 101–417.

42. Vanhaeren M, d' Errico F. Aurignacian ethno-linguistic geography of Europe revealed by personal ornaments. J Archaeol Sci. 2006; 33: 1–24.
